# Metabolomics reveal drought-stress responses in guayule, a semi-arid rubber crop

**DOI:** 10.1007/s11306-026-02487-5

**Published:** 2026-06-16

**Authors:** Huy Phan, Hussein Abdel-Haleem

**Affiliations:** US Arid Land Agricultural Research Center, USDA-Agricultural Research Services, Maricopa, AZ 85138 USA

**Keywords:** Drought response, Guayule, Isoprenoid metabolism, Metabolomics, Rubber biosynthesis

## Abstract

**Introduction:**

Natural rubber is crucial for many industries, and guayule, (Parthenium argentatum A. Gray, is a promising alternative source for rubber in the arid and semi-arid regions. Rubber concentration production in guayule increases under drought stress, but the metabolic reasons remain unclear.

**Objectives and Methods:**

Using untargeted metabolomic approach, this study analyzed drought-induced metabolomic changes in two guayule cultivars, AZ-4 and CAL-2, focusing on key metabolites linked to resin and rubber biosynthesis.

**Results:**

We identified 53 drought-responsive metabolites, with 27 differing significantly between cultivars. AZ 4 showed increased stress signaling molecules and precursor metabolites, indicating rapid metabolic activation under drought, while CAL 2 accumulated triterpenoid saponins and lysophospholipids, suggesting membrane stabilization and long term osmoprotective defense. Under drought, both cultivars shared depletion of 4'-phosphopantothenoylcysteine, indicating a potential bottleneck in Coenzyme A biosynthesis. Reduced ubiquinone-1 further suggested a diversion of isoprenoid precursors away from respiration and toward secondary metabolism. This shift is linked to activation of the MVA and MEP pathways, which enhanced flux into rubber and resin biosynthesis rather than ubiquinone production. Although cis-1,4-polyisoprene was not directly measured, the observed increases in key terpenoid precursors and pathway‑associated metabolites are consistent with enhanced flux toward rubber biosynthesis under drought conditions.

**Conclusion:**

Divergent metabolic patterns reflect genetic and biochemical diversity: AZ 4 favors rapid stress signaling and precursor synthesis, whereas CAL 2 emphasizes antioxidant and osmoprotective mechanisms. These biomarkers make them valuable for breeding drought-tolerant, and high-yield guayule germplasm to advance sustainable rubber production in dry regions.

**Supplementary Information:**

The online version contains supplementary material available at https://doi.org/10.1007/s11306-026-02487-5.

## Introduction

Guayule is a desert plant capable of producing high-value rubber (cis-1,4-polyisoprene). Unlike *Hevea brasiliensis* (Wild. Ex A. Juss.) Mull. Arg., guayule thrives in semi-arid regions with minimal irrigation requirements (Elshikha, et al., 2023; Hunsaker & Elshikha, 2017). In its native region, guayule can grow on 250–380 mm of annual rainfall (Bekaardt, et al., 2010). Luo and Abdel-Haleem (2019) reported that reducing irrigation amounts resulted in lower biomass, total rubber and resin yields. The variation in reduction among guayule genotypes indicate wide genetic and phenotypic diversities among guayule natural population that include cultivars and advanced germplasm from different gene pools and wild accessions. Guayule’ growing conditions outside tropical climates, making it a promising model for studying stress-induced rubber biosynthesis (Abdel-Haleem, et al., 2019; Cherian, et al., 2019). Drought stress stimulates secondary metabolism and redirects carbon flux toward rubber production, revealing trade-offs between growth and biosynthetic investment (Dong, et al., 2021; Nelson, et al., 2019). Guayule’s complex genome and well-characterized pathways support multi-omics integration for mechanistic studies (Nelson, et al., 2019). Guayule also synthesizes diverse terpenoids, flavonoids, and phenolics, providing biochemical markers for abiotic stress response (Dong, et al., 2021; Luo, et al., 2020).

Guayule genotypes exhibit genetic differences that influence drought‑responsive mechanisms, and prior studies provide general insights into these processes. Despite these differences, research on guayule as a species has shown that intrinsic genetic traits help maintain core drought‑responsive mechanisms. This includes modulation of the mevalonate (MVA) and methylerythritol phosphate (MEP) pathway fluxes, ensuring a degree of consistency in drought response across genotypes (Dong, et al., 2021; Nelson, et al., 2019).

Key metabolites in guayule, including lysyl-phenylalanine, kiwiionoside, camellianin A, catechin 3’-glucuronide, azukisaponin III, and licoricesaponins, provide critical insights into how the MVA and MEP pathways operate under drought conditions to enhance rubber biosynthesis in multiple rubber-producing species, including *Hevea brasiliensis, Taraxacum brevicorniculatum, and Aspergillus nidulans*, (Cherian, et al., 2019; Chow, et al., 2012; Miziorko, 2011; Yamashita & Takahashi, 2020; Yamashita, et al., 2016). Lysyl-phenylalanine and kiwiionoside function as precursors and intermediates feeding into the MVA pathway, suggesting increased flux toward isoprenoid and triterpenoid production (Yamashita & Takahashi, 2020). Phenolic compounds like camellianin A (Lidya Cahyo Bawono 2023) and catechin derivatives (Singh, 2025), serve dual roles as antioxidants and modulators of phenylpropanoid metabolism, indicating coordination between stress defense and energy allocation. Resins like triterpenoid saponins, including azukisaponin III, act as both protective metabolites and indicators of MVA pathway activity (Kundu, et al., 2025; Yao, et al., 2020) in model plants such as *Arabidopsis thaliana* and *Nicotiana benthamiana*, and medicinal plants, such as *Panax ginseng* (Yu-Nan, et al., 2014), *Glycyrrhiza glabra* (Li, et al., 2016), and *Medicago truncatula* (Nelson, 2009), reflecting enhanced biosynthetic throughput under drought. The presence of 4’-phosphopantothenoylcysteine suggests a conserved checkpoint regulating Coenzyme A availability, which may control acetyl-CoA entry into both MVA and MEP pathways (Soto, et al., 2011). Meanwhile, iridoid glycosides and lysophospholipids hint at plastidial MEP activity and membrane remodeling contribute to drought stress adaptive responses (Wang, et al., 2024). Collectively, these metabolites provide a biochemical signature that allows prediction of pathway fluxes and prioritization of rubber and terpenoid biosynthesis.

Guayule cultivars, developed for different growth conditions, exhibit both similarities and notable differences, influenced by distinct genetic backgrounds as well as environmental factors Genotypic variability among guayule genotypes could affect key traits such as abiotic stress tolerance, secondary metabolite production, and rubber biosynthesis, leading to genotype-specific regulation of metabolic pathways, including the synthesis of antioxidants and triterpenoids, which in turn result in characteristic metabolic profiles. While genetic differences play a central role, environmental conditions in the respective growing regions further modulate genotypic responses. The U.S. Southwestern’ s semi-arid environment, origin center of guayule, characterized by higher temperatures, lower humidity, and more pronounced seasonal drought (Abdel-Haleem, et al., 2019; Foster, et al., 2011). These conditions place strong physiological demands on plants, potentially intensifying stress responses and shaping patterns of metabolic adaptation. In contrast, California’s desert-adjacent and coastal regions often experience milder temperature fluctuations and slightly higher soil moisture levels, which may favor vegetative growth over stress-induced metabolite accumulation (Bañuelos, et al., 2022; Placido, et al., 2021). These environmental factors influence the balance between rubber biosynthesis and the production of secondary metabolites such as antioxidants and triterpenoids, contributing to the metabolic distinctions observed between cultivars. As growing conditions, irrigation practices, seasonal variability and soil structure influence growth and rubber yield (Abdel-Haleem, et al., 2018) it is reasonable to comparing different guayule genotypes, for example Arizona and California cultivars under uniform soil and environmental conditions to identify candidate genes and stable biochemical pathways involved in drought stress response. Understanding these interactions can guide the selection of parental lines optimized for specific growing conditions, while sustaining high rubber yield and stress tolerance.

This study pursues two complementary objectives. First, it aims to identify metabolites involved in drought signaling and defense by characterizing the stress-responsive metabolomic profiles of two different guayule cultivars (AZ-4 was developed for Arizona’ growing conditions, and CAL-2 was developed for California growing conditions). This analysis will reveal key metabolites and biochemical pathways activated in response to drought stress, shedding light on how guayule senses and responds to water deficit. Second objective aims to study metabolites directly related to resin and rubber biosynthesis. By comparing metabolomic differences between AZ-4 and CAL-2, the research will explore how cultivar-specific biochemical traits correspond to variations in rubber yield, informing breeding strategies to develop drought-tolerant and high-yielding guayule germplasm.

## Materials and methods

### Plant material and growth conditions

Two guayule cultivars, AZ-4 (PI59967) (Ray et al., 1999) and CAL-2 (PI478667) (Tysdal et al., 1983), were selected based on their contrasting responses to reduced irrigation (Luo & Abdel-Haleem, 2019). AZ-4 and CAL-2 are tetraploid lines developed for Arizona and California growing conditions, respectively. One-year-old transplanted plants of each cultivar were sampled from three replicated plots across two irrigation trials: well-irrigated (IR) and water-stressed, or drought, (D), conducted at the Maricopa Agricultural Center, University of Arizona (33°03′55’’ N, 111°58′31’’ W). The soil at the trial site was classified as Casa Grande series (fine-loamy, mixed, hyperthermic Typic Natragrids). Both trials were furrow-irrigated bi-weekly during plant establishment. Prior to tissue collection, irrigation was withheld for three months in the water-stressed trial, while the well-irrigated plots continued to receive water every 2–3 weeks, adjusted for weather conditions. The experiment was conducted using a randomized complete block design consisting of three blocks within a single field site. Each block contained one irrigated (IR) plot and one drought‑reduced (D) plot, resulting in three biological replicates per treatment. The IR and D plots within each block were positioned adjacent to one another to ensure that all plots experienced the same environmental conditions, including soil characteristics, microclimate, and sunlight exposure. This spatial arrangement minimized environmental confounding and allowed irrigation treatment to be isolated as the primary experimental factor. Bark tissues from the upper third of the stem were harvested from each plot, representing biological replicates for both IR and DR conditions. Samples were immediately immersed in liquid nitrogen and stored at − 80 °C for downstream metabolomic analysis.

Guayule plants were grown under the designated irrigation treatments, and tissue samples were collected from three independent biological replicates per treatment group. Each biological replicate corresponded to a separate field plot (for example, DR_AZ4‑R1, DR_AZ4‑R2, and DR_AZ4‑R3). Within each plot, stem bark tissue was harvested from 3 individual plants, which were pooled to generate a single composite sample representing that plot. All biological replicates were processed independently through sample preparation, metabolite extraction, and LC–MS analysis. No technical replicates were used for statistical comparisons; all downstream analyses were performed using these three independent biological replicates per treatment (n = 3).

### Instrumentation, reagents and chemicals

Metabolite profiling and compound identification were performed using a Vanquish Flex Ultra-Performance Liquid Chromatography (UPLC) system coupled with a Q Exactive Plus Mass Spectrometer (Thermo Fisher Scientific, USA). Chromatographic separation was achieved using an ACQUITY UPLC HSS T3 column (100 mm × 2.1 mm, 1.8 μm; Waters Corporation). Centrifugation steps were carried out using a temperature-controlled functional centrifuge (Eppendorf, Germany) to maintain sample integrity during preparation. All solvents and reagents were of LC–MS grade. Acetonitrile, methanol, and formic acid were purchased from Merck (Germany) and used without further purification. Mobile phases were freshly prepared and filtered through 0.22 μm membranes prior to use.

### Sample preparation

Frozen bark tissue samples in Sect. 2.1 were thawed prior to extraction. Approximately 100 mg of each sample was weighed into a microcentrifuge tube and extracted with 800 μL of 80% methanol. For samples weighing less than 100 mg, the extraction volume was adjusted proportionally at a rate of 8 μL of 80% methanol per mg of tissue. Each sample was vortexed for 30 s, followed by sonication for 30 min at 4 °C to enhance metabolite release. Samples were then stored at − 20 °C for 1 h, vortexed again for 30 s, and incubated at 4 °C for 30 min. After incubation, samples were centrifuged at 12,000 rpm for 15 min at 4 °C. The resulting supernatant was transferred to a fresh tube and subjected to a second round of − 20 °C storage for 1 h, followed by centrifugation at 12,000 rpm for 15 min at 4 °C.

For LC–MS analysis, 200 μL of the final supernatant was combined with 5 μL of DL-o-Chlorophenylalanine (0.14 mg/mL) as an internal standard and transferred to autosampler vials. To assess analytical reproducibility, QC samples were prepared by pooling equal volumes of extract from all individual samples. The pooled QC sample underwent the same preparation protocol as described above and was analyzed alongside experimental samples. Five pairwise comparisons were conducted to evaluate metabolic differences across cultivars and irrigation conditions: analysis 1: Water-stressed (DR) vs. Well-irrigated (IR) samples; analysis 2: AZ-4 vs. CAL-2 cultivars; analysis 3: CAL-2_DR vs. CAL-2_IR, analysis 4: AZ-4_DR vs. AZ-4_IR; analysis 5: AZ-4_DR vs. CAL-2_DR.

### Ultra-performance liquid chromatography and mass spectrometry analysis

Chromatographic separation was carried out using a Vanquish Flex UPLC system coupled with a Q Exactive Plus Mass Spectrometer (Thermo Fisher Scientific, USA) equipped with electrospray ionization mass spectrometry (ESI–MS). The liquid chromatography utilized an ACQUITY UPLC HSS T3 column measuring 100 mm by 2.1 mm with a particle size of 1.8 μm, which provided high-resolution separation of analytes. The mobile phase consisted of solvent A, 0.05% formic acid in water, and solvent B, acetonitrile, operated under a gradient elution program starting at 5% solvent B for the first minute, followed by a linear increase to 95% solvent B over 11 min. This was maintained at 95% solvent B from 12 to 13.5 min before quickly returning to 5% solvent B by 13.6 min and re-equilibrating until 16 min. The flow rate was maintained at 0.3 mL per minute, with the column temperature set at 40 °C and the sample manager kept at 4 °C. Mass spectrometry data were collected using full scan mode over an m/z range of 70 to 1050 at a resolution of 70,000, along with data-dependent MS/MS acquisition targeting the top 10 most intense ions at a resolution of 17,500, employing higher-energy collisional dissociation. In positive ion mode (ESI +), the instrument was set with a heater temperature of 300 °C, sheath gas flow of 45 arbitrary units, auxiliary gas flow of 15, sweep gas flow of 1, spray voltage of 3.0 kV, capillary temperature of 350 °C, and an S-Lens RF level of 30%. For negative ion mode (ESI–), similar parameters were used except the spray voltage was set at 3.2 kV and the S-Lens RF level at 60%. This setup ensured sensitive and accurate detection of metabolites under both ionization modes.

### Metabolite identifications and statistical analysis

Metabolite identification was performed by Novogene using their standard LC–MS untargeted metabolomics workflow, and confidence levels were assigned according to the Metabolomics Standards Initiative (MSI) guidelines. Metabolites confirmed with authentic reference standards, requiring matching accurate mass, retention time, and MS/MS fragmentation patterns, were classified as MSI Level 1. Metabolites annotated based on accurate mass (mass error < 5 ppm) together with MS/MS spectral similarity to public databases including HMDB, MassBank, and ChemSpider were designated as MSI Level 2. Features showing characteristic fragment ions consistent with a known chemical class but lacking sufficient evidence for unique structural assignment were categorized as MSI Level 3, while unidentified features without interpretable spectra were considered MSI Level 4 and excluded from biological interpretation. Although MSI levels were not explicitly assigned, the metabolite identification output includes several independent quality metrics that together provide a clear indication of identification confidence. Accurate mass information (m/z, molecular weight, monoisotopic mass) combined with low mass error values (Δppm typically < 5 ppm) supports high confidence putative identifications consistent with MSI Level 2 criteria. Additional fields such as adduct type, isotope similarity, fragmentation score, and spectral similarity scores from mzCloud and mzVault further strengthen the reliability of the annotations by confirming the presence of expected isotopic patterns and MS/MS fragment ions. These combined metrics allow metabolites to be classified as putatively identified when strong mass accuracy and spectral matches are present, and as confirmed when matched to authentic standards. Thus, while MSI levels are not explicitly listed, the dataset provides sufficient confidence indicators for readers to assess the reliability of each reported metabolite.

Statistical analysis was conducted to identify significant metabolic differences across sample groups. Raw mass spectrometry data were first acquired and aligned using Compound Discoverer 3.0 (Thermo Fisher Scientific), based on the mass-to-charge ratio (m/z) and retention time of ion signals. Ion features from both positive (ESI +) and negative (ESI −) electrospray ionization modes were merged and imported into SIMCA-P software (version 14.1) for multivariate statistical analysis. An initial Principal Component Analysis (PCA) was performed as an unsupervised method to visualize overall data structure and detect potential outliers. To further explore group-specific differences, supervised regression modeling was applied using Partial Least Squares Discriminant Analysis (PLS-DA) and Orthogonal Partial Least Squares Discriminant Analysis (OPLS-DA). These models were used to identify potential biomarkers that distinguish between experimental conditions.

Significant metabolites were selected using a combination of univariate statistics (t‑test, p < 0.05) and multivariate importance metrics (VIP > 1.5). Because this study used an untargeted LC–MS workflow with a large number of detected features, we recognize that multiple‑testing correction (e.g., FDR adjustment) is commonly applied to control for false positives. In this analysis, FDR correction was not implemented because the primary goal was to identify broad metabolic patterns and candidate biomarkers rather than to perform strict hypothesis testing on each individual feature. However, we acknowledge that the absence of multiple‑testing correction may increase the likelihood of type I errors, and we have therefore interpreted the identified metabolites as putative rather than definitive markers.

## Results

### Metabolomic adjustments and identification of key drought-responsive metabolites in guayule

To investigate metabolomic adjustments in guayule under drought versus irrigated conditions, samples from two cultivars (AZ-4 and CAL-2) were collected and processed separately to preserve cultivar-specific variation. For comparative purposes, metabolomic data were subsequently integrated and analyzed using high-resolution mass spectrometry. Structural assignments were based on accurate mass measurements and MS/MS fragmentation data, supported by online databases (HMDB, ChemSpider, MassBank), and validated against authentic standards when necessary.

Metabolomic profiling detected 1038 metabolites in positive ion mode and 889 in negative ion mode, totaling 1836 unique metabolites, with 91 overlapping between modes. Table 1 summarizes the top metabolites with significant fold changes and p-values. Drought stress led to significant increases in saponins such as licoricesaponin-J2 and momordin I, while compounds, such as 4’-phosphopantothenoylcysteine were consistently downregulated. Hierarchical clustering analysis (HCA) identified 43 statistically significant metabolites (Supplementary Fig. 1), revealing distinct clustering patterns between drought and irrigated samples. The top five upregulated metabolites included proline and monoacylglycerol (MG)(20:3) (Table 1). Notably, only the biological metabolite 4’-phosphopantothenoylcysteine, was shared between the raw fold-change and HCA analyses, showing consistent depletion under drought.

**Table 1:** Top five metabolites between drought and irrigated guayule via two analyses

Top Metabolites	Analysis	Log2(Fold Change)	− Log10(p-value)
2-dodecylbenzenesulfonic acid	Raw	− 3.27	1.28
7a,12a-dihydroxy-3-oxo-4-cholenoic acid		− 3.04	0.41
Lenalidomide		− 2.68	1.75
4’-phosphopantothenoylcysteine		− 2.64	2.49
Catechin 3’-glucuronide		− 2.44	0.70
Licoricesaponin-J2		+ 4.50	0.70
Momordin I		+ 4.42	0.60
Licoricesaponin-B2		+ 4.38	0.73
Oleragenoside		+ 4.38	0.68
Azukisaponin III		+ 4.38	0.58
Lenalidomide	Hierarchical Cluster Analysis	− 2.68	1.75
4’-phosphopantothenoylcysteine		− 2.64	2.49
Salviaflaside		− 2.16	1.33
4-trimethylammoniobutanoic acid		− 1.84	1.39
Stizolobic acid		− 1.83	1.34
Monoacylglycerol(MG)(20:3(6,8,11)-OH(5)/0:0/0:0)		+ 3.80	1.64
Dihydrolipoamide		+ 2.58	1.61
PIP(PGD2/18:0)		+ 2.36	1.62
Proline		+ 2.10	1.66
Cornoside		+ 1.96	2.81

( −) sign notes for depletion and ( +) sign notes for accumulation; yellow: metabolites overlapped between two analyses. Full list of metabolites can be found in Supplementary Table 9

To identify key metabolic responses to drought stress, we curated three ranked lists: the top 80 most significant, most accumulated, and most depleted metabolites], out of approximately 1,836 detected compounds. These lists were derived from both pooled and cultivar-specific comparisons of drought versus irrigated conditions. By integrating results from AZ-4 and CAL-2 independently, as well as from a combined analysis, Table 2 summarizes metabolites consistently responsive to drought across genotypes and comparisons. The overlap among these three lists yielded 53 unique drought-responsive metabolites, representing a focused set of candidates for downstream pathway enrichment and functional interpretation.

**Table 2:** Twenty‑four drought‑responsive metabolites selected from 1927 significant features detected in positive and negative ion mode

Metabolite	Category	Log2(Fold Change)	Description
4’-phosphopantothenoylcysteine	Precursors of Resin and Rubber Biosynthesis	− 3.78	Precursor to Coenzyme A, essential for fatty acid and isoprenoid biosynthesis
Arginine		+ 3.65	Amino acid involved in nitrogen metabolism; may support precursor synthesis
Monoacylglycerol(MG)(20:3-OH/0:0/0:0)		+ 3.80	Lipid precursor that may contribute to membrane or rubber-related lipid synthesis
Ornithine		+ 3.84	Urea cycle intermediate, precursor to polyamines and nitrogen donors
Proline		+ 2.1	Amino acid with osmoprotective properties; supports biosynthesis under stress
Ubiquinone-1		− 2.07	Isoprenoid derivative structurally related to polyisoprenes; may share biosynthetic origin
Azukisaponin III	Product, Byproduct, or Parallel Product of Resin and Rubber Biosynthesis	+ 4.81	Saponin found in latex-producing plants; may be co-extracted with rubber
Licoricesaponin-B2		+ 5.11	Saponin from licorice, often found in latex-rich plants
Licoricesaponin-J2		+ 4.5	Similar to Licoricesaponin-B2; triterpenoid saponin
Masticadienonic acid		− 1.19	Triterpenoid resin acid found in latex-producing plants
Momordin I		+ 4.87	Triterpenoid saponin associated with latex-rich plant species
Oleragenoside		+ 5.34	Iridoid glycoside similar to oleoside; latex-related
13-HDoHE	Stress Signaling Molecules	+ 2.4	Hydroxylated derivative of docosahexaenoic acid (DHA); lipid mediator involved in oxidative stress and inflammation
Cornoside		+ 1.96	Iridoid glycoside with anti-inflammatory and stress-related activity
LysoPC(18:3/0:0)		− 1.05	Lysophospholipid; involved in membrane remodeling and stress signaling
Methyl 7-epi-12-hydroxyjasmonate glucoside		+ 1.45	Jasmonate derivative; key plant stress hormone
Methyl salicylate O-[rhamnosyl-(1- > 6)-glucoside]		+ 1.64	Salicylate conjugate; plant defense signal
PIP(PGD2/18:0)		+ 2.36	Prostaglandin D2-containing lipid; immune and stress mediator
4’-methyl-epigallocatechin 7-glucuronide	Antioxidant and Defense	+ 2.09	Flavonoid conjugate with antioxidant properties
9-methyluric acid		− 5.73	Derivative of uric acid; retains antioxidant activity
Camellianin A		+ 1.95	Flavonoid glycoside with strong antioxidant activity
Coniferin		+ 1.14	Lignan precursor with antioxidant and structural roles
Feruloylquinic acid		+ 1.44	Ester of ferulic acid; antioxidant and UV-protective
Uric acid		+ 3.14	Antioxidant that scavenges reactive oxygen species

Metabolites were curated into four functional categories (precursors/resin, saponins/resins, stress signaling, antioxidants/defense), with six representative compounds per group chosen for biological relevance to guayule drought response and rubber biosynthesis. Synthetic or exogenous contaminants were excluded. ( −) indicates depletion under drought, while ( +) indicates accumulation. Functional descriptions were obtained from the Plant Metabolic Network (Hawkins et al., 2025) and RefMetaPlant (Shi et al., 2024). The full list of 53 metabolites is provided in Supplementary Table 7

Pathway enrichment via correlation network analysis revealed 35 significantly enriched pathways, with the top five involving valine, leucine, and isoleucine degradation; purine metabolism; pyruvate metabolism; and arginine and proline metabolism (Table 3).

**Table 3:** Top five enriched pathways between drought and irrigated guayule via Correlation Network Analysis

Top Pathways	Analysis	− Log10(p-value)
Valine, Leucine and Isoleucine Degradation	Correlation network analysis	7.19
Purine Metabolism		6.52
Pyruvate Metabolism		6.47
Arginine and Proline Metabolism		6.33
Steroidogenesis		5.85

Full list of pathways can be found in Supplementary Table 9

### Cultivar-specific metabolomic responses and genotype-level drought stress specific signatures in AZ-4 and CAL-2

To investigate how AZ-4 and CAL-2 adjusted their metabolomes under drought stress, metabolomic profiles of guayule plants grown under drought conditions were compared to those cultivated under irrigation.

For AZ-4, the top metabolites exhibited significant fold changes and p-values. Drought stress induced notable upregulation of amino acids such as ornithine and arginine, alongside bioactive compounds like cyclomulberrin and monoacylglycerol (MG)(20:3) (Supplementary Table 1). Conversely, biological metabolites including spinacoside D, and prostaglandin lactone-diol were markedly downregulated. HCA identified 23 statistically significant metabolites, with 4’-phosphopantothenoylcysteine consistently emerging as the most depleted compound under drought conditions. Among the top upregulated metabolites, methyl salicylate O-[rhamnosyl-(1 → 6)-glucoside] and 5-p-coumaroylquinic acid showed strong statistical support (Supplementary Table 1). Interestingly, no overlaps were observed among the top five metabolites between raw fold-change and HCA analyses. Pathway enrichment analysis for AZ-4 revealed significant activation in coenzyme production and carbohydrate remodeling, with pantothenate and CoA biosynthesis [− log10(p-value) = 3.35] and amino sugar metabolism (2.87) showing strong enrichment (Supplementary Table 2).

For CAL-2, drought conditions triggered pronounced upregulation of triterpenoid saponins such as oleragenoside, licoricesaponin B2, and momordin I, alongside lipid mediators like 13-HDoHE and dihydrolipoamide. In contrast, metabolites including 9-methyluric acid, and 7a,12a-dihydroxy-3-oxo-4-cholenoic acid were downregulated. HCA also demonstrated consistent depletion of 4’-phosphopantothenoylcysteine across both raw and clustered datasets. Notably, biological metabolites, like 13-HDoHE, and dihydrolipoamide, exhibited strong statistical significance with − log10(p-values) exceeding 1.60 (Supplementary Table 1). Pathway enrichment analysis for CAL-2 revealed distinct metabolic reprogramming under drought, characterized by elevated activity in redox homeostasis, amino acid catabolism, and nitrogen salvage. Ubiquinone biosynthesis [− log10(p-value) = 4.21] was prominently enriched (Supplementary Table 2), alongside beta-alanine metabolism, phenylalanine and tyrosine metabolism, ammonia recycling, and cysteine metabolism (4.00 each).

To further dissect genotype-specific metabolic responses, we applied the ranked-list approach, top 80 most significant, most accumulated, and most depleted metabolites, to each cultivar independently. This targeted analysis yielded a refined set of 27 unique metabolites (Table 4), representing cultivar-specific signatures of drought. These compounds were selected for their consistent presence across multiple ranking criteria.

**Table 4:** Twenty-four filtered metabolites from AZ-4 versus CAL-2 based on significance and fold changes

Metabolite	Category	Log2(Fold Change)	Description
3-pyridylacetic acid	Precursors of Rubber Biosynthesis	+ 4.76	A small organic acid that may participate in nitrogen metabolism linked to secondary biosynthesis
5-imino-2-methyl-1-cyclopenten-1-ol		+ 6.84	A cyclopentene derivative potentially involved in early terpenoid or isoprene unit formation
Histidylaspartic acid		− 6.56	A peptide possibly involved in enzymatic regulation or precursor signaling
Kiwiionoside		− 7.47	A glycoside that may be part of upstream metabolic flux toward rubber biosynthesis
Lysyl-phenylalanine		+ 7.30	A dipeptide that could contribute to amino acid-derived biosynthetic intermediates
Mono(glucosyluronic acid)bilirubin	Product, Byproduct, or Parallel Product of Rubber Biosynthesis	+ 2.35	A bilirubin conjugate that could be excreted alongside rubber-related metabolic waste
Monoacylglycerol(MG)(0:0/5-iso PGF2VI/0:0)		+ 3.10	A lipid signaling molecule that may arise as a byproduct of polyisoprene metabolism
PIP(PGF1alpha/16:2)		+ 7.35	A prostaglandin-like compound possibly co-produced during lipid-based biosynthesis
Prostaglandin lactone-diol		− 6.81	A cyclic prostaglandin derivative that may reflect parallel lipid processing in rubber-producing tissues
Rheinoside C		− 6.5	A plant glycoside that may accumulate in latex-rich tissues as a secondary metabolite
2-aminophenol N-formate sulfate	Stress Signaling Molecules	+ 9.92	A nitrogen-containing compound possibly involved in oxidative or pathogen-triggered signaling
7’-O-methylmarmin		+ 7.60	A coumarin derivative that may act as a signal in response to biotic or abiotic stress
Citrusin B		+ 2.20	A limonoid involved in plant defense and stress-induced secondary metabolism
Mirificin		− 7.11	A phytoestrogen-like compound that could modulate hormonal or environmental stress responses
Sampatrilat		+ 3.42	A vasopeptidase inhibitor that may mimic or interfere with peptide-based stress signaling
4’-methyl-epigallocatechin 7-glucuronide	Antioxidant and Defense	− 7.2	A flavonoid glucuronide with strong antioxidant and anti-inflammatory activity
6-dehydrotestosterone glucuronide		+ 6.17	(Not previously listed—please confirm if this belongs in your dataset.)
6-methoxyluteolin 7-glucuronide		+ 4.79	A methoxylated flavone that helps mitigate oxidative stress in plant tissues
Calendoflaside		− 2.26	A saponin-like compound that enhances plant immunity
Chalcomoracin		+ 3.10	A prenylated flavonoid with antimicrobial and antioxidant effects
Isoeriocitrin		− 1.85	A citrus-derived flavonoid with potent radical-scavenging properties
Kaempferol 3-rhamnosyl-(1- > 3)-(4’’’-acetylrhamnosyl)(1- > 6)-glucoside		+ 5.76	A complex flavonoid glycoside contributing to pathogen resistance
Myricanol 5-[arabinosyl-(1- > 6)-glucoside]		+ 2.28	A glycosylated diarylheptanoid with anti-inflammatory and defense roles
Ononin		− 3.66	An isoflavone glycoside with antioxidant and protective functions

Metabolites were curated into four functional categories (precursors/resin, saponins/resins, stress signaling, antioxidants/defense), with six representative compounds per group chosen for biological relevance to guayule drought response and rubber biosynthesis. Synthetic or exogenous contaminants were excluded. ( −) sign notes for higher concentration in CAL-2 and ( +) sign notes for higher concentration in AZ-4. Descriptions obtained from Plant Metabolic Network (Hawkins et al., 2025) and RefMetaPlant (Shi et al., 2024). The full list of 27 metabolites is provided in Supplementary Table 8

Among the 27 cultivar-specific metabolites identified, the majority were classified under Category 4: Antioxidant and Defense (33.33%), reflecting a strong genotype-level emphasis on stress mitigation. Equal proportions (18.52%) were assigned to Category 1: Precursors of Resin and Rubber Biosynthesis, Category 2: Products/Byproducts of Rubber Biosynthesis, and Category 3: Stress Signaling Molecules, highlighting potential links between drought adaptation and specialized secondary metabolism. Notably, no metabolites fell into Category 5: Plant-Derived but Uncharacterized, and a smaller fraction (11.11%) were categorized as Category 6: True Synthetic & Contaminants, suggesting a refined and biologically relevant subset for cultivar-specific pathway analysis.

### Metabolite comparisons between AZ-4 and CAL-2 plants grown under drought conditions

To evaluate and compare side-by-side metabolomic differences between AZ-4 and CAL-2 under drought, samples from each drought cultivar were collected for metabolomic profiling. Supplementary Table 3 highlights the top metabolites with significant fold changes and statistical relevance. In AZ-4, 2-aminophenol N-formate sulfate was identified as the most dominant metabolite [log2(fold change) = 9.82; − log10(p-value) = 1.80], indicating a strong cultivar-specific accumulation. Additional metabolites such as PIP(PGF1alpha/16:2), lysyl-phenylalanine, and 5-imino-2-methyl-1-cyclopenten-1-ol showed elevated levels with –log10(p-values) ranging from 1.48 to 2.68, demonstrating metabolites only found in AZ-4, not CAL-2. Top metabolites from CAL-2 under drought stress were examined for fold change and statistical significance (Supplementary Table 3). Among the most enriched compounds, 4’-Methyl-epigallocatechin 7-glucuronide exhibited the highest accumulation [log2(fold change) = 7.20; − log10(p-value) = 1.93], followed closely by kiwiionoside (7.17; 1.56) and mirificin (7.11; 1.40). Additional compounds such as prostaglandin lactone-diol (6.81; 0.92) and paucin (6.57; 1.52) also showed elevated levels compared to AZ-4 under drought conditions.

HCA of two cultivars under drought stress identified a metabolite profile identical to that obtained through raw data analysis, reaffirming the robustness of the observed metabolic shifts (Supplementary Table 2, 3). Key metabolites found only in AZ-4 such as 2-aminophenol N-formate sulfate, PIP(PGF1alpha/16:2), lysyl-phenylalanine, and 5-imino-2-methyl-1-cyclopenten-1-ol remained consistently elevated, supporting their relevance in AZ-4’s drought-induced metabolic response. Furthermore, HCA between two cultivars under drought stress produced a metabolite profile for CAL-2 almost identical to that obtained through raw data analysis (raw yielded prostaglandin lactone-diol, HCA yielded rheinoside C). Metabolites such as 4’-methyl-epigallocatechin 7-glucuronide, kiwiionoside, mirificin, and paucin remained consistently elevated, reinforcing the reproducibility of CAL-2’s metabolic response and highlighting stable accumulation patterns across analytical approaches. HCA confirmed almost identical metabolite profiles to raw analysis, reinforcing consistent drought-induced shifts in metabolites for AZ-4 and CAL-2 side-by-side.

Side-by-side pathway enrichment analysis of AZ-4 and CAL-2 under drought stress revealed overlapping activation across nine metabolic pathways (Supplementary Table 4). Estrone metabolism showed the highest enrichment [− log10(p-value) = 3.48], followed by tyrosine metabolism (3.06), ubiquinone biosynthesis (2.92), and tryptophan metabolism (2.81). Additional pathways including arginine and proline metabolism (2.68), pyrimidine metabolism (2.68), amino sugar metabolism (2.43), pantothenate and CoA biosynthesis (1.99), and steroidogenesis (1.72) were also enriched, indicating consistent metabolic shifts across both cultivars.

### Exploring chemical compounds associated with resin and rubber biosynthesis under drought stress conditions

Across all comparisons, regardless cultivars, 1836 metabolites were detected and categorized into six functional groups: (1) precursors of resin and rubber biosynthesis, (2) products, byproducts, or parallel products of resin and rubber biosynthesis, (3) stress signaling molecules, (4) antioxidants and defense compounds, (5) plant-derived but uncharacterized metabolites, and (6) true synthetics and contaminants. This classification framework enables targeted interpretation of metabolite roles, distinguishing those involved in stress adaptation from those contributing to specialized secondary metabolism. Organizing the data this way enhances biological relevance and facilitates downstream pathway mapping. The six functional metabolite categories were defined based on established biochemical pathways in guayule and other terpenoid‑producing plants, combined with known roles of specific metabolites in central carbon metabolism, precursor supply, and downstream rubber‑related pathways. Compounds were assigned to categories such as “precursors” or “products of rubber biosynthesis” based on their documented biochemical functions, pathway annotations in KEGG and HMDB, and prior literature on guayule metabolism. In cases where direct pathway placement was not possible—such as metabolites upstream of multiple branches or compounds with ambiguous roles—the assignments were treated as inferred classifications intended to aid interpretation rather than definitive biochemical placements. Additionally, features annotated as synthetic or pharmaceutical compounds (e.g., lenalidomide, ribavirin) were considered misannotations or background signals arising from database matching and were therefore excluded from biological analysis and from pathway‑level interpretation. This clarification ensures that only biologically plausible metabolites contribute to the functional grouping and subsequent discussion.

**Fig. 1:**
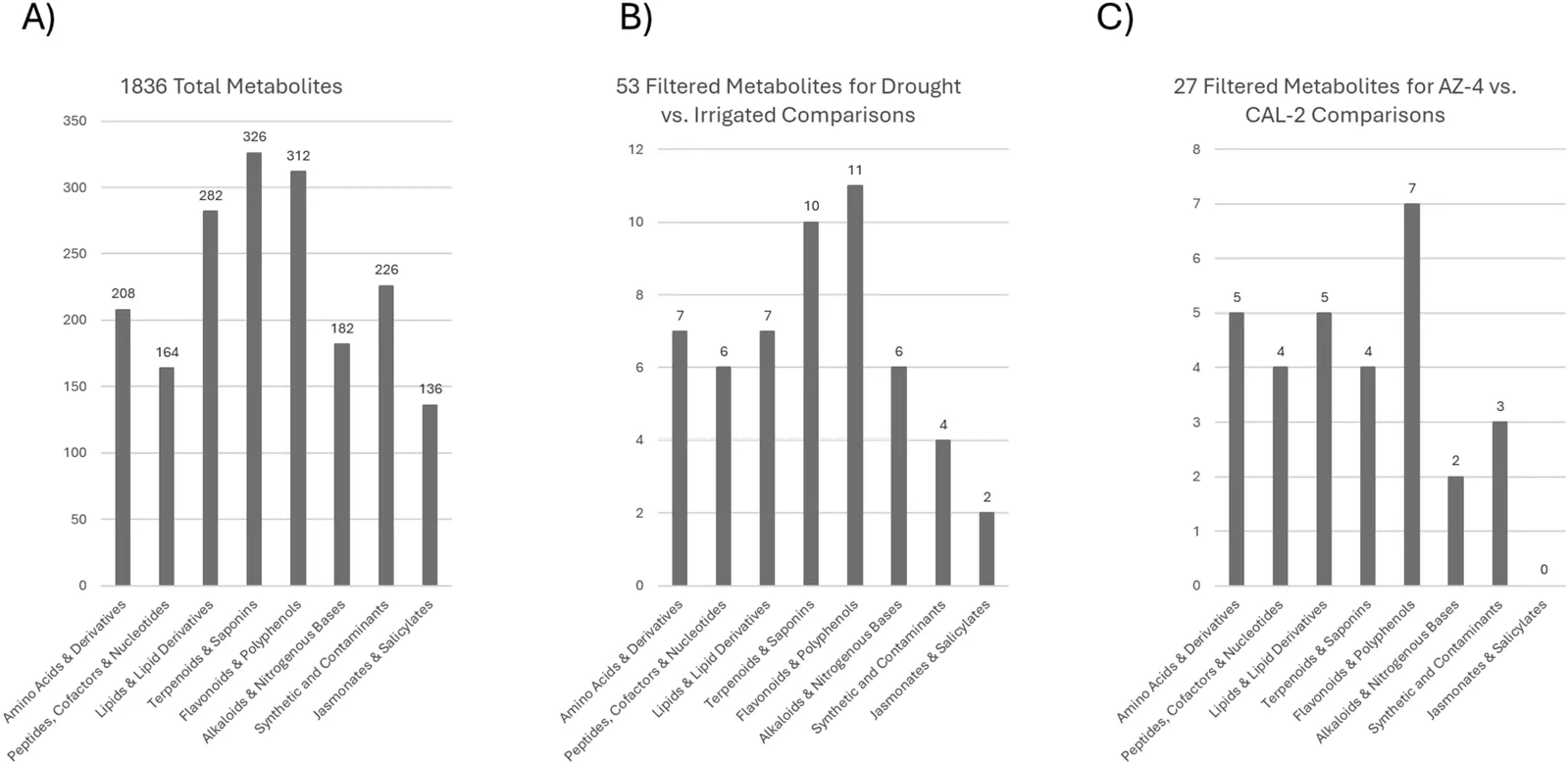
Distribution of metabolites into pathway categories. **A** 1836 metabolites categorized into 6 pathway categories. **B**. 53 filtered metabolites from drought versus irrigated conditions based on significance and fold changes into 6 pathway categories. **C** 27 filtered metabolites from AZ-4 versus CAL-2 based on significance and fold changes into 6 pathway categories

Among these metabolites, 437 metabolites were identified as potentially influencing resin or rubber biosynthesis, either as precursors, environmental signaling molecules, or direct byproducts. Narrowing the focus further, 230 metabolites fell within the product, byproduct, or parallel product category of rubber biosynthesis (Fig. 1A; Supplementary Table 14). However, since these metabolites did not consistently appear across the three key lists of most significant, most accumulated, and most depleted metabolites (Sect. 3.1), selecting the 53 overlapping metabolites provides a more precise and fine-tuned set for investigating chemical compounds associated with resin and rubber biosynthesis under stress conditions. These 53 candidate metabolites were categorized using the same six functional groups, reinforcing our approach to enhance biological insight and support pathway mapping under stress.

The broad metabolite pool detected (1836 compounds) shows relatively even distribution across categories, with the highest proportions in (4) Antioxidant and Defense (25.00%) and (6) True Synthetic & Contaminants (24.85%), and a notable presence of (5) Plant-Derived but Uncharacterized metabolites (19.78%) (Fig. 1A). In contrast, the focused set of 53 prioritized metabolites is enriched in (3) stress signaling molecules (26.42%) and (4) antioxidant and defense (26.42%), along with increased representation of (1) precursors (20.75%) and (2) products/byproducts (18.87%) related to resin and rubber biosynthesis (Fig. 1B). Importantly, none of the selected metabolites fell into the (5) plant-derived but uncharacterized category, and fewer were classified as (6) true synthetic & contaminants (7.55%), reflecting a biologically meaningful refinement towards pathways central to drought response and specialized biosynthesis of resin and rubber. This shift in distribution highlights the biological specificity of the identified metabolite set and its associations with stress adaptation and specialized metabolic pathways.

### Metabolite comparisons between AZ-4 and CAL-2 regardless of treatment conditions

To further characterize the metabolomic differences between AZ-4 and CAL-2 under both conditions, a refined analysis was conducted (Supplementary Table 5), that showed top five most significantly enriched metabolites. For AZ-4, notably, 2-aminophenol N-formate sulfate exhibited the highest fold change at 9.86 with a − log10(p-value) of 3.66, marking it as the most prominent compound in this profile. Other metabolites with elevated levels included 7’-O-methylmarmin at 7.21 (2.64), PIP(PGF1alpha/16:2) at 7.12 (2.20), and lysyl-phenylalanine at 7.00 (2.10). In contrast to AZ-4, the metabolomic profile of CAL-2 under drought stress revealed a distinct set of enriched compounds. Kiwiionoside showed the highest accumulation with a log2(fold change) of 7.33 and a − log10(p-value) of 3.54, followed by mirificin at 7.06 (2.69). Other notable metabolites included 4’-methyl-epigallocatechin 7-glucuronide at 6.82 (1.71), rheinoside C at 6.41 (2.55), and histidylaspartic acid at 6.26 (2.21).

HCA was conducted to further validate the metabolomic distinctions between AZ-4 and CAL-2 regardless of exposure. Metabolites such as 2-aminophenol N-formate sulfate, and 7’-O-methylmarmin continued to cluster tightly and showed up in AZ-4, while kiwiionoside, mirificin, and rheinoside C formed a distinct cluster associated with only CAL-2. As both approaches led to identical sets of top five metabolites, this reinforced the separation between the two cultivars, confirming that each exhibits a unique metabolic response.

To explore the differences between AZ-4 and CAL-2, pathway enrichment analysis for two cultivars regardless of stress highlighted significant enrichment of a total of 17 pathways. Top five pathways yielded two to be highly relevant in AZ-4: tryptophan metabolism in AZ-4, with a strong correlation network score of 6.57, and tyrosine metabolism also showed notable enrichment in AZ-4, scoring 5.86 (Supplementary Table 6), indicating these pathways play prominent roles in this cultivar’s metabolome. The other three pathways of the top five list are for CAL-2, with strong enrichment of ubiquinone biosynthesis, with a correlation score of 7.86. Estrone metabolism also showed significant enrichment at 7.25, followed by amino sugar metabolism at 6.19. These results indicate that these pathways are prominently involved in CAL-2, and not AZ-4. Interestingly, all these top five pathways are also present in the drought-specific metabolomic differences when comparing AZ-4 and CAL-2 in Supplementary Table 4.

## Discussion

### Statistical and biological integration identifies key drought-responsive metabolites in guayule cultivars

**Fig. 2:**
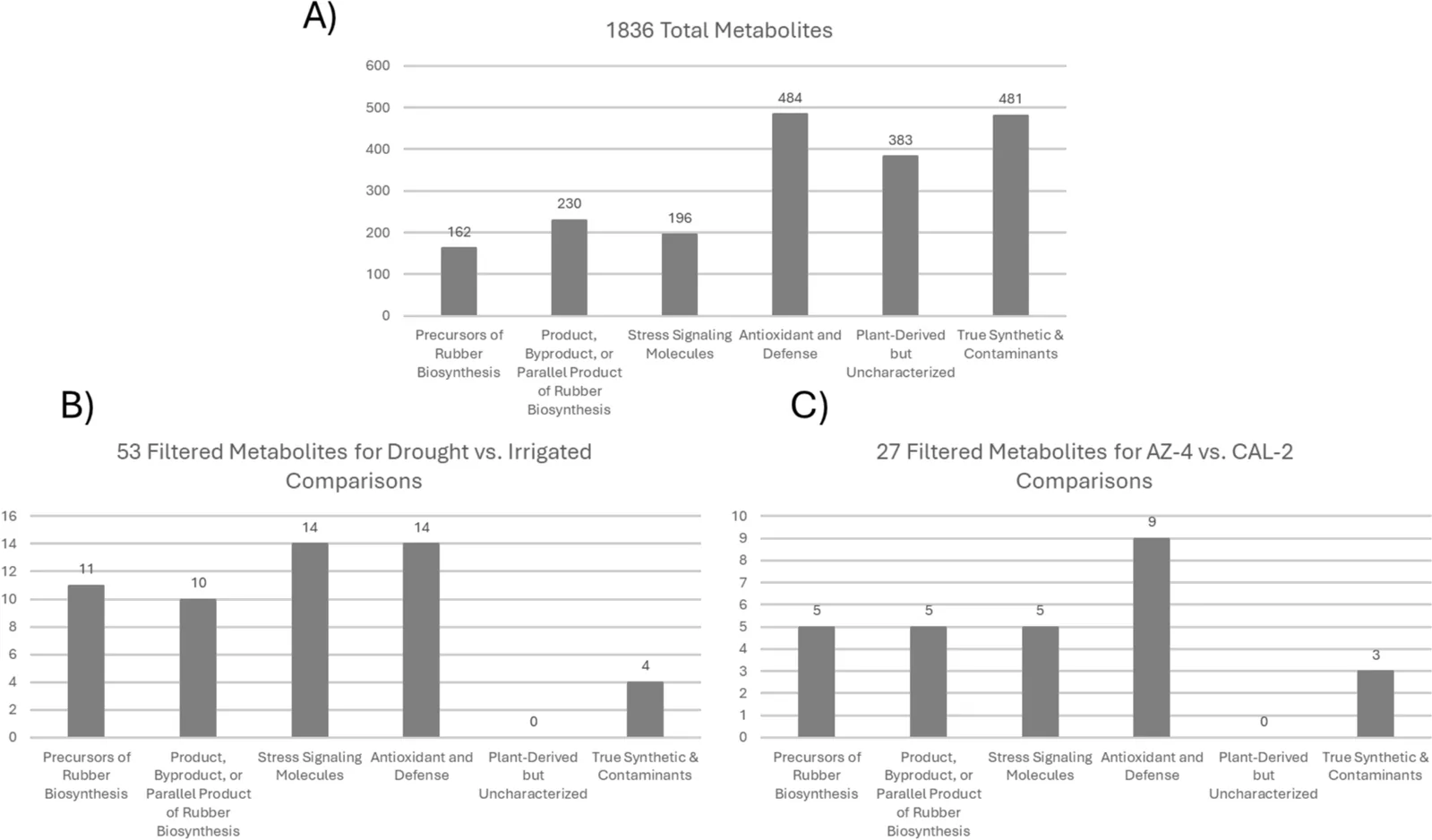
Distribution of metabolites into biochemical categories. **A**. 1836 metabolites categorized into 8 biochemical categories. **B**. 53 filtered metabolites from drought versus irrigated conditions based on significance and fold changes into 8 biochemical categories. **C** 27 filtered metabolites from AZ-4 versus CAL-2 based on significance and fold changes into 8 biochemical categories

To identify drought-responsive metabolites in guayule cultivars AZ-4 and CAL-2, we applied a dual filtering approach based on statistical significance (− log10p-value) and magnitude of change (log2fold change) (Steuer, et al., 2007; Ren, et al. 2015; Li, 2020), selecting compounds that were both significantly altered and biologically relevant (Fig. 1A, D). This strategy mirrors approaches used in other metabolomic studies, where similar filtering criteria have proven effective in revealing meaningful drought-associated metabolic shifts (Ackah, et al. 2021; Wang, et al., 2024; Wonneberger, et al., 2025). Our process narrowed the full metabolomic dataset to 53 metabolites of interest, which were subsequently organized into functional categories to facilitate interpretation. These included 7 amino acids and derivatives, 6 peptides, cofactors, and nucleotide-related compounds, 7 lipids and lipid derivatives, 10 terpenoids and saponins, 11 flavonoids and polyphenols, 6 alkaloids and nitrogenous bases, 2 jasmonates and salicylates, 4 true synthetic or contaminant compounds, and no plant-derived and uncharacterized (Fig. 2A, B). Despite the reduction in dataset size, the filtering approach preserved a broad spectrum of metabolite classes, ensuring that chemical diversity was maintained across primary and secondary metabolic pathways (Fig. 1B, Table 2). The final set of 53 metabolites spans key functional categories, including, capturing both core metabolic shifts and cultivar-specific adaptations. This balance between statistical rigor and biological relevance allowed for a focused yet representative view of guayule’s drought-responsive metabolome, without over-filtering potentially informative compounds.

To determine whether AZ-4 and CAL-2 are sufficiently distinct in their metabolomic profiles, a targeted comparison was conducted under irrigation, drought, and regardless of conditions. A filtering approach was applied to select metabolites showing both statistical significance (− log10p-value) and biologically meaningful fold changes (log2fold change), narrowing the dataset to 27 key metabolites that capture core biochemical differences between the two cultivars while preserving functional diversity. The filtered compounds included 5 amino acids and derivatives, 4 peptides, cofactors, and nucleotide-related metabolites, 5 lipids and lipid derivatives, 4 terpenoids and saponins, 7 flavonoids and polyphenols, 2 alkaloids and nitrogenous bases, and 3 true synthetic or contaminant compounds, with no jasmonates or salicylates retained, and no plant-derived and uncharacterized (Fig. 2A, C). This approach ensured that critical metabolite classes representing stress signaling, rubber biosynthesis, and antioxidant/defense functions were retained, potentially allowing for a concise yet informative comparison across environmental conditions. By focusing on these 27 metabolites, the analysis highlights cultivar-specific biochemical strategies and identifies candidate compounds associated with stress tolerance mechanisms and useful for breeding programs targeting productivity and drought tolerance. Overall, this analysis supports the hypothesis that AZ-4 and CAL-2 possess sufficiently distinct metabolomic traits that could evolve using the selection of those cultivars and could be the foundation to develop advanced guayule cultivars.

### Biochemical divergence in guayule cultivars

Comparative metabolomic analysis of guayule cultivars AZ-4 and CAL-2 regardless of conditions revealed consistent biochemical divergence, with each cultivar exhibiting distinct adaptive metabolic strategies. Regardless of growth conditions, AZ-4 showed elevated levels of rubber biosynthesis precursors, particularly lysyl-phenylalanine (S. tables 6, 7, 9), along with stress-signaling molecules such as 7′-O-methylmarmin, indicating active carbon allocation toward biosynthetic processes and metabolic flexibility (Duangngam, et al., 2020; Liu, et al., 2017). In contrast, CAL-2 accumulated higher levels of antioxidant and defense-related metabolites, including mirificin (Liu, et al., 2018) and 4′-methyl-epigallocatechin 7-glucuronide ( (Stalmach, et al., 2009) (Tables 2, 4; S. tables 7, 9), as well as rubber-pathway co-products like rheinoside C (Xu, et al., 2024), suggesting a strategy centered on cellular stability and defense readiness. Under drought stress, these differences became more pronounced. AZ-4 retained strong stress-signaling capacity, supported by compounds such as sampatrilat (Ramlal, et al. 2022), and continued to express rubber biosynthesis activity despite water limitation ((Yamashita & Takahashi, 2020). CAL-2, meanwhile, intensified its antioxidant and defense chemistry, with elevated levels of mirificin and 4′-methyl-epigallocatechin 7-glucuronide, and showed signs of osmoprotective adaptation through amino acid–linked pathways. Clustering analysis reinforced these intrinsic biochemical differences and aligned with the individual irrigated and drought datasets. AZ-4 was consistently enriched in stress-signaling molecules, including sampatrilat, citrusin B (Li, et al., 2023), and 2-aminophenol N-formate sulfate, as well as rubber precursors like lysyl-phenylalanine and kiwiionoside (Duangngam, et al., 2020). CAL-2, on the other hand, showed a distinct accumulation of antioxidants and defense compounds such as mirificin, chalcomoracin ((Hu, et al., 2025), and 4′-methyl-epigallocatechin 7-glucuronide (Table 2; S. table 6). These patterns suggest a potential genetic basis for metabolite accumulation, as related genes were previously identified (Dong, et al., 2021), supporting the hypothesis that AZ-4’s metabolism favors rapid biosynthesis and stress signaling, whereas CAL-2 emphasizes long-term defense and oxidative protection. The distinct and consistent metabolomic profiles of AZ-4 and CAL-2 highlight their divergent physiological strategies and underscore the biochemical complexity underlying guayule’s response to environmental conditions.

Argentatins and guayulins are well known specialized metabolites in guayule, but they were not detected in the dataset. This outcome is consistent with the extraction and chromatographic conditions used in this study. The workflow employed an 80% methanol aqueous extraction, which is optimized for polar and semi polar metabolites but is not efficient for highly hydrophobic terpenoids such as argentatins and guayulins, which are typically enriched in resin fractions and require non polar or biphasic extraction solvents (e.g., hexane, MTBE, chloroform) for reliable recovery. In addition, the reverse phase LC–MS method used here is tuned for broad metabolite coverage but does not provide optimal retention or ionization for these non polar sesquiterpenes. As a result, their absence reflects methodological limitations rather than true biological absence.

### Pathway-specific metabolomic shifts in guayule under drought stress

To reveal drought-responsive metabolic pathways in guayule cultivars AZ-4 and CAL-2, side-by-side comparisons were conducted using raw metabolomic profiling and hierarchical clustering analysis. By integrating both methods, we gained a more comprehensive and dependable understanding of how each cultivar reconfigures its metabolism under drought stress, revealing distinct and biological strategies for adaptation to arid conditions.

**Fig. 3:**
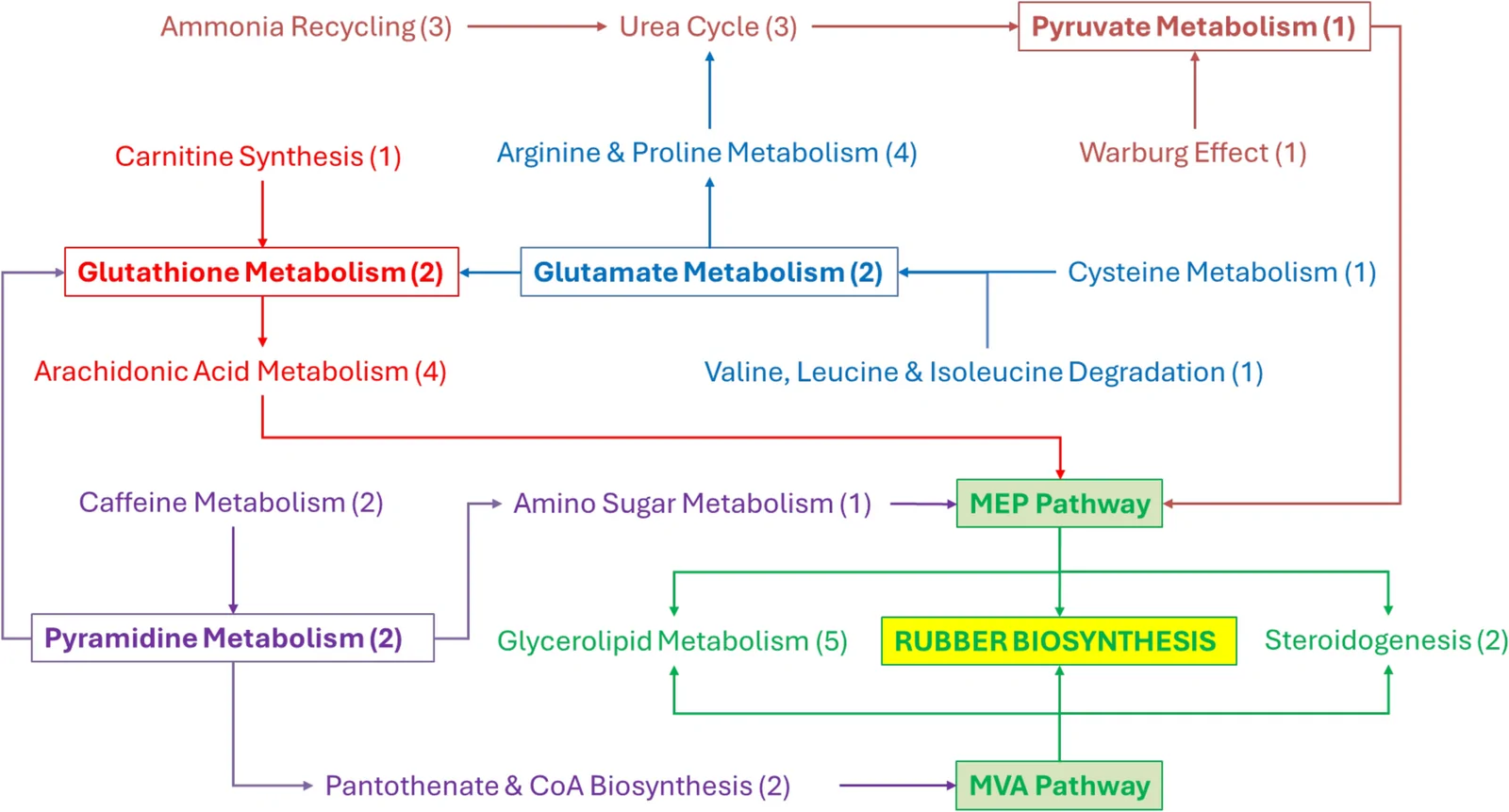
Significant pathways from 53 filtered metabolites from drought versus irrigated conditions based on significance and fold changes into 5 biosynthesis networks. **A** Amino acid metabolism with Glutamate metabolism (in bold) as hub. **B** Energy and carbon metabolism with Pyruvate metabolism (in bold) as hub. **C** Stress signaling and defense with Glutathione metabolism (in bold) as hub. **D** Nucleotide and cofactor metabolism with Purine metabolism and Pyramidine metabolism (in bold) as hub. **E** Rubber biosynthesis and specialized pathways with MVA pathway and MEP pathway (in bold) as hub

**Fig. 4:**
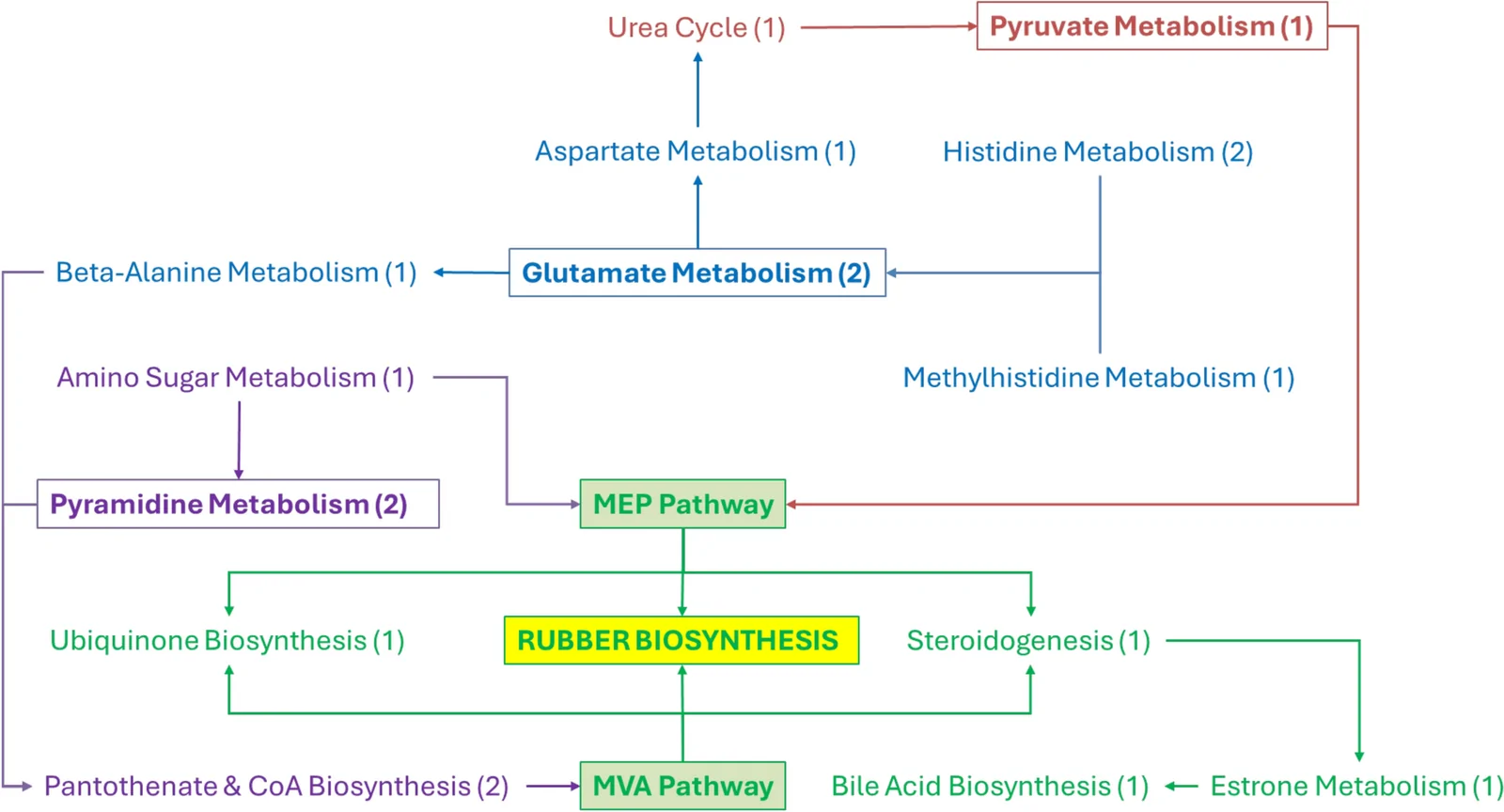
Significant pathways from 27 filtered metabolites from AZ-4 versus CAL-2 based on significance and fold changes into 4 biosynthesis networks. **A** Amino acid metabolism with Glutamate metabolism (in bold) as hub. B Energy and carbon metabolism with no pathways as hub. **C** Nucleotide and cofactor metabolism with Purine metabolism and Pyramidine metabolism (in bold) as hub. **E** Rubber biosynthesis and specialized pathways with MVA pathway and MEP pathway (in bold) as hub

A key finding was the consistent depletion of 4’-phosphopantothenoylcysteine in both AZ-4 and CAL-2 under drought and irrigated conditions. As a direct intermediate in the pantothenate and Coenzyme A (CoA) biosynthesis pathway (Genschel, 2004; Sasaki & Nagano, 2004), its reduction suggests a conserved metabolic response aimed at downregulating energy-intensive biosynthetic processes (Fig. 3E; Fig. 4A, C). This interpretation is supported by transcriptomic studies in the drought tolerant plant species Polygonatum kingianum, where drought stress significantly reduced the expression of genes involved in pantothenate and CoA biosynthesis, alongside other high-energy metabolic routes (Qian, et al., 2021). Additionally, proteomic studies in tea plants under drought revealed downregulation of several CoA-dependent enzymes involved in fatty acid biosynthesis (Gu, et al., 2020), suggesting that plants may suppress CoA-utilizing pathways to conserve energy under water-limited conditions. In this study, the consistent depletion of 4’-phosphopantothenoylcysteine across cultivars and conditions may reflect a similar adaptive mechanism, positioning this metabolite as a promising target for future research into gene regulation and drought tolerance.

Building on this shared metabolic signature, further cultivar-specific differences emerged in how AZ-4 and CAL-2 modulate their metabolomes under drought stress. In AZ-4, drought stress led to the accumulation of 5-p-coumaroylquinic acid, and methyl salicylate O-[rhamnosyl-(1 → 6)-glucoside], as well as feruloylquinic acid and coniferin, all derived from the phenylalanine and tyrosine metabolism pathway (Gao, et al., 2023; König, et al., 2014; Vogt, 2010; Yoo, et al., 2013). These phenolic compounds are associated with lignin biosynthesis (Le Roy, et al., 2017), oxidative stress buffering (Clifford, et al., 2017), and systemic acquired resistance (Chen, et al., 2019; Klessig, et al., 2018), indicating a prioritization of structural reinforcement and long-distance stress signaling in AZ-4’s drought response. Additional accumulation of flavonoids such as camellianin A and catechin 3’-glucuronide in AZ-4 further supports its antioxidant-driven strategy to mitigate drought-induced oxidative stress (Bao & Fenwick, 2004; Liu, et al., 2010).

Interestingly, CAL-2 exhibited a consistent accumulation of triterpenoid saponins, including licoricesaponin-B2, licoricesaponin-J2, and azukisaponin III. These metabolites are known for their roles in stress signaling, membrane stabilization, and defense against abiotic stress (Augustin et al., 2011), particularly under drought conditions where they may reinforce cellular structures, modulate membrane fluidity, and mitigate oxidative damage (Nasrollahi, et al., 2014; Amnay, et al. 2023). CAL-2 also showed increased levels of lysophospholipids such as LysoPC(18:3/0:0) and LysoPE(18:3/0:0), which are associated with membrane remodeling and may contribute to maintaining cellular integrity under water deficit (Calvano, et al. 2020; Gai, et al., 2020; Shen, et al., 2022). Additionally, CAL-2 showed marked depletion of 9-methyluric acid, a methylated derivative of uric acid and the terminal product of purine metabolism, reflecting a potential suppression of purine catabolism, which in plants is closely tied to nitrogen recycling (Brychkova, et al., 2008) and nucleotide turnover (Werner & Witte, 2011), and often downregulated under drought to conserve energy (Li, et al., 2025; Thu & Tegeder, 2025).

These pathway-level differences identifies specific metabolic hubs that differentiate drought adaptation strategies in AZ‑4 and CAL‑2. In particular, enrichment of phenylpropanoid‑derived antioxidants and salicylate‑linked signaling metabolites in AZ-4 suggests enhanced engagement of glutathione‑associated redox pathways, which connect nitrogen metabolism to oxidative stress buffering. Conversely, CAL‑2’s accumulation of triterpenoid saponins and lysophospholipids points to modulation of pyruvate‑linked carbon allocation and membrane remodeling processes, metabolic hubs not previously highlighted in guayule drought research. These findings reveal cultivar‑specific metabolic architectures that may influence precursor availability for rubber‑related isoprenoid biosynthesis or stress resilience, providing novel insight into how drought alters core metabolic networks in guayule.

Overall, this dual comparison, marked by the accumulation of phenolic and antioxidant compounds in AZ-4 and protective triterpenoid saponins alongside the depletion of purine catabolites in CAL-2, highlights distinct pathway-level strategies in guayule’s drought adaptation (Fig. 2C, D). The cultivar AZ-4 favors biosynthesis of phenylpropanoid-derived protective phenolics and antioxidants, while CAL-2 may engage in metabolic restraint and membrane remodeling, with the shared reduction of 4’-phosphopantothenoylcysteine across both cultivars pointing to a core drought-responsive mechanism centered on CoA-linked energy metabolism.

### Drought stimulates MVA and MEP pathways and isoprenoid flux toward resin and rubber biosynthesis in guayule

Drought stress is widely recognized as a stimulus for increased rubber concentration (rubber per unit biomass) production in guayule (Luo & Abdel-Haleem, 2019; Veatch-Blohm, et al., 2006), and this response can be explained through a combination of metabolic and physiological mechanisms (Fig. 2A–D). Under water deficit conditions, plants experience oxidative stress and shifts in hormonal signaling, particularly involving jasmonates and abscisic acid (Siddiqi & Husen, 2019; Wang, et al., 2020; Yang, et al., 2019). These signals are known to activate the mevalonate (MVA) and methylerythritol phosphate (MEP) pathways; (Chevalier, et al. 2024; Singh, et al., 2024) (Fig. 2A–C), which produce isopentenyl pyrophosphate (IPP) and dimethylallyl pyrophosphate (DMAPP), the universal precursors for both cis-1,4-polyisoprene (rubber) and a wide range of terpenoid-based secondary metabolites (Wang, et al., 2019). The MVA pathway in the cytosol primarily supports the biosynthesis of triterpenoids and rubber, while the MEP pathway in plastids contributes to monoterpenes and iridoid glycosides (Sawai & Saito, 2011; Singh, et al., 2024; Wang, et al., 2019). Metabolomic data revealed increased levels of triterpenoid saponins and related compounds under drought, including azukisaponin III (28-fold), momordin I (29-fold), and licoricesaponins (35-fold for B2; 23-fold for J2). These metabolites are biosynthetically parallel to rubber, sharing IPP as a precursor (Kundu, et al., 2025; Yang, et al., 2022), and their accumulation suggests increased flux through the isoprenoid biosynthetic network.

Additional upregulation of cyclomulberrin (15-fold), a triterpenoid derivative, further supports enhanced activity of the MVA pathway under drought. Interestingly, masticadienonic acid, a triterpenoid intermediate, was significantly depleted by half, which may reflect its short-lived role and rapid conversion into downstream products (Arrieta, et al., 2003; Giner-Larza, et al., 2002), consistent with elevated flux through rubber-related biosynthesis. Similarly, 4’-phosphopantothenoylcysteine, a precursor in coenzyme A biosynthesis and upstream of acetyl-CoA entry into the MVA pathway (Genschel, 2004; Oliver, et al., 2009; Sasaki & Nagano, 2004), was reduced by approximately 93%, potentially reflecting a bottleneck or regulatory shift favoring downstream triterpenoid and rubber biosynthesis.

The MEP pathway also showed signs of activation under drought, similar to published studies (Banerjee & Sharkey, 2014; Perreca, et al., 2020). Notably, oleoside dimethyl ester and oleragenoside, both iridoid glycosides derived from the MEP pathway, were upregulated by 22-fold and 39-fold, respectively, indicating enhanced plastidial isoprenoid activity. This observation aligns with transcriptomic data from guayule, which reported significant upregulation of iridoid glycosides under drought conditions (Dong, et al., 2021). Conversely, ubiquinone-1, a mitochondrial electron carrier synthesized from IPP (Eubel, et al., 2004; Fedor, et al., 2017), was downregulated fourfold in drought guayule This downregulation suggests a potential rerouting of IPP away from respiratory quinone biosynthesis and toward the production of secondary metabolites, agreeing with the finding of drought-driven metabolic prioritization favoring rubber and triterpenoid synthesis genes (Dong, et al., 2021). Together, these metabolite dynamics highlight a drought-induced metabolic strategy in guayule that not only activates key biosynthetic genes in the MVA and MEP pathways (Fig. 3E; Fig. 4D) but also modulates precursor availability and flux directionality within the isoprenoid network, ultimately favoring rubber and triterpenoid biosynthesis.

Although the MVA and MEP pathways are central to rubber biosynthesis, key intermediates such as IPP and DMAPP were not detected in our untargeted LC–MS dataset. This is expected because IPP/DMAPP are highly polar diphosphate esters present at very low steady state levels and are rarely observed in standard reverse phase metabolomics workflows without specialized extraction, ion pairing chromatography, or targeted MRM methods. Therefore, the interpretation of increased flux through the MVA and MEP pathways is based on coordinated changes in upstream and downstream metabolites rather than direct measurement of IPP/DMAPP. Additionally, cis-1,4-polyisoprene, the final product of rubber biosynthesis, is not directly detectable in standard metabolomics workflows due to its polymeric and hydrophobic nature (Kawahara, 2023), the presence and upregulation of these co-products serve as indirect indicators of increased flux through rubber-related pathways (Chow, et al., 2012). The data suggest that guayule may be channeling more carbon into isoprenoid metabolism via the two MVA and MEP pathways during drought, potentially as a protective strategy. Rubber itself may function as a carbon sink when growth is suppressed, and its inert, hydrophobic properties could contribute to cellular stability under desiccation stress (Nizami, et al., 2014; Ma, et al. 2024; Zhang, et al., 2024).

**Fig. 5:**
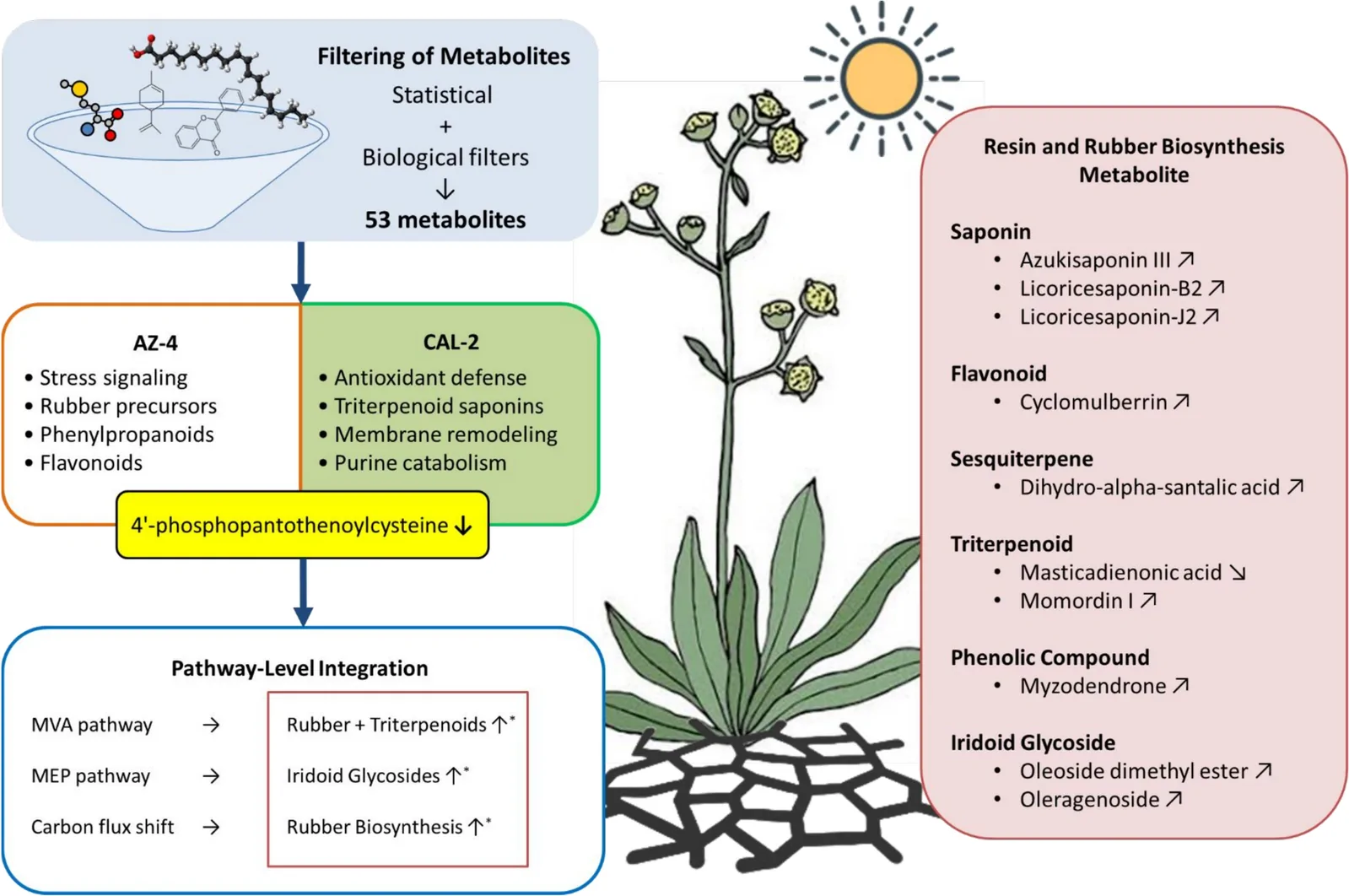
Graphical abstract summarizing the physiological pathways and metabolomic profiling of two guayule cultivars, AZ-4 and CAL-2, under drought stress. Untargeted LC–MS analysis identified approximate 1836 metabolites, which were filtered to highlight key compounds associated with drought signaling, defense responses, and biosynthetic pathways for resin and rubber. The schematic illustrates differential accumulation of stress-related metabolites and pathway-specific intermediates, linked to the mevalonate (MVA) and methylerythritol phosphate (MEP) pathways. Comparative mapping reveals cultivar-specific metabolic fluxes, with AZ-4 favoring stress-induced secondary metabolism and CAL-2 exhibiting enhanced rubber biosynthesis under moderate drought. Additionally, metabolites in the functional group resin and rubber biosynthesis metabolite genuinely accumulated

Field studies have consistently shown higher rubber yields in guayule under drought (Elshikha, et al., 2023; Hunsaker, et al., 2019; Luo & Abdel-Haleem, 2019; Veatch-Blohm, et al., 2006; Wang, et al., 2022), and transcriptomic analyses have reported upregulation of key biosynthetic genes, including cis-prenyltransferases and rubber particle-associated proteins (Dong, et al., 2021). The metabolite shifts observed in this study align with these findings, reinforcing the hypothesis that drought not only activates stress signaling but also enhances the plant’s capacity to produce rubber. In this context, the accumulation of metabolites, either byproducts or parallel products of rubber biosynthesis via the two MVA and MEP pathways, serve as biochemical signatures of a drought-induced metabolic state of guayule favorable to rubber production (Figure 5).

### Future directions

Building on the metabolomic insights from this study, future research could aim to unravel the regulatory networks controlling key drought-responsive metabolites, particularly those consistently altered cultivars growing under drought conditions. The distinct metabolite profiles of AZ-4 and CAL-2, highlighting rapid stress signaling versus long-term antioxidant defense, provide a framework for dissecting genotype-specific adaptation strategies. Expanding the metabolomic profiling additional guayule accessions and wild relatives could uncover novel drought-adaptive compounds and broaden the genetic base for breeding. Environmental metabolomics, tracking changes over time and across different lengths of drought, may also identify dynamic biomarkers signaling early drought response or recovery potential. Integrating transcriptomic and proteomic data and metabolomic profiles could clarify how these biochemical traits are encoded and expressed under different environmental conditions. Functional validation of key metabolites, such as kiwiionoside and mirificin, through gene editing or overexpression studies, would further elucidate their roles in stress tolerance and rubber biosynthesis, and reveal how they influence growth, rubber yield, and survival under drought.

## Conclusion

This study had two primary goals: 1) to characterize drought-induced metabolomic profiles of two guayule cultivars AZ-4 and CAL-2 focusing on MVA and MEP pathway metabolites linked to resin and rubber biosynthesis; and 2) to determine whether distinct metabolomic signatures correlate with different genetic backgrounds. Using a rigorous filtering approach, 53 drought-responsive metabolites were identified and 27 different key metabolites between two cultivars. AZ-4 exhibited increased stress-signaling molecules and phenylpropanoid antioxidants, indicating rapid metabolic activation under drought. CAL-2 showed accumulation of resins like triterpenoid saponins, and lysophospholipids, reflecting membrane stabilization and higher baseline long-term defense. Both cultivars shared a notable depletion of 4’-phosphopantothenoylcysteine, suggesting a conserved metabolic checkpoint regulating Coenzyme A biosynthesis.

Drought stimulated activation of the MVA and MEP pathways, increasing flux toward isoprenoid metabolites linked to rubber and triterpenoid resin synthesis. Elevated triterpenoid saponins and iridoid glycosides confirm enhanced isoprenoid metabolism. The reduction in ubiquinone-1 hinted at resource reallocation from respiration to secondary metabolite production. Though cis-1,4-polyisoprene itself is not directly measurable, related metabolite accumulation supports increased rubber biosynthesis under drought. The metabolomic divergence between AZ-4 and CAL-2 suggests genetic differences and biochemical plasticity. AZ-4 prioritizes rubber and resin precursor production and rapid stress signaling, while CAL-2 emphasizes antioxidant defenses and osmoprotection. These complementary traits support their use as parent lines for breeding drought-tolerant, high-yielding hybrids. Future work should integrate multi-omics data to clarify regulatory networks and functionally validate key metabolites like kiwiionoside and mirificin. Evaluating hybrid lines between AZ-4 and CAL-2 will reveal the potential for combined beneficial traits. Expanding surveys to other guayule accessions with different responses to drought stress (Luo & Abdel-Haleem, 2019) may uncover new drought-adaptive metabolites. Environmental metabolomics could also identify early biomarkers of stress response. Overall, this work advances understanding of guayule’s metabolic reprogramming under drought. It highlights how MVA and MEP pathways coordinate increased rubber biosynthesis. These insights provide valuable targets for molecular breeding and biotechnological innovation. Ultimately, they support developing guayule cultivars capable of maintaining productivity in water-limited climates.

## Supplementary Information

Below is the link to the electronic supplementary material.

Supplementary file1 (PDF 1033 KB)

Supplementary file2 (DOCX 37 KB)

Supplementary file3 (XLSX 828 KB)

Supplementary file4 (XLSX 641 KB)

Supplementary file5 (XLSX 966 KB)

Supplementary file6 (XLSX 716 KB)

Supplementary file7 (XLSX 944 KB)

Supplementary file8 (XLSX 70 KB)

## Data Availability

The raw metabolomics data generated in this study are available upon request.
