# Supplementary material for: Metabolomics reveal drought-stress responses in guayule, a semi-arid rubber crop
Source: Metabolomics. 2026 Jun 16;22(4):97. doi: 10.1007/s11306-026-02487-5 (PMC13272222; doi:10.1007/s11306-026-02487-5)
Supplement: Supplementary file 1 — Supplementary file1 (PDF 1033 KB) [file 11306_2026_2487_MOESM1_ESM.pdf]

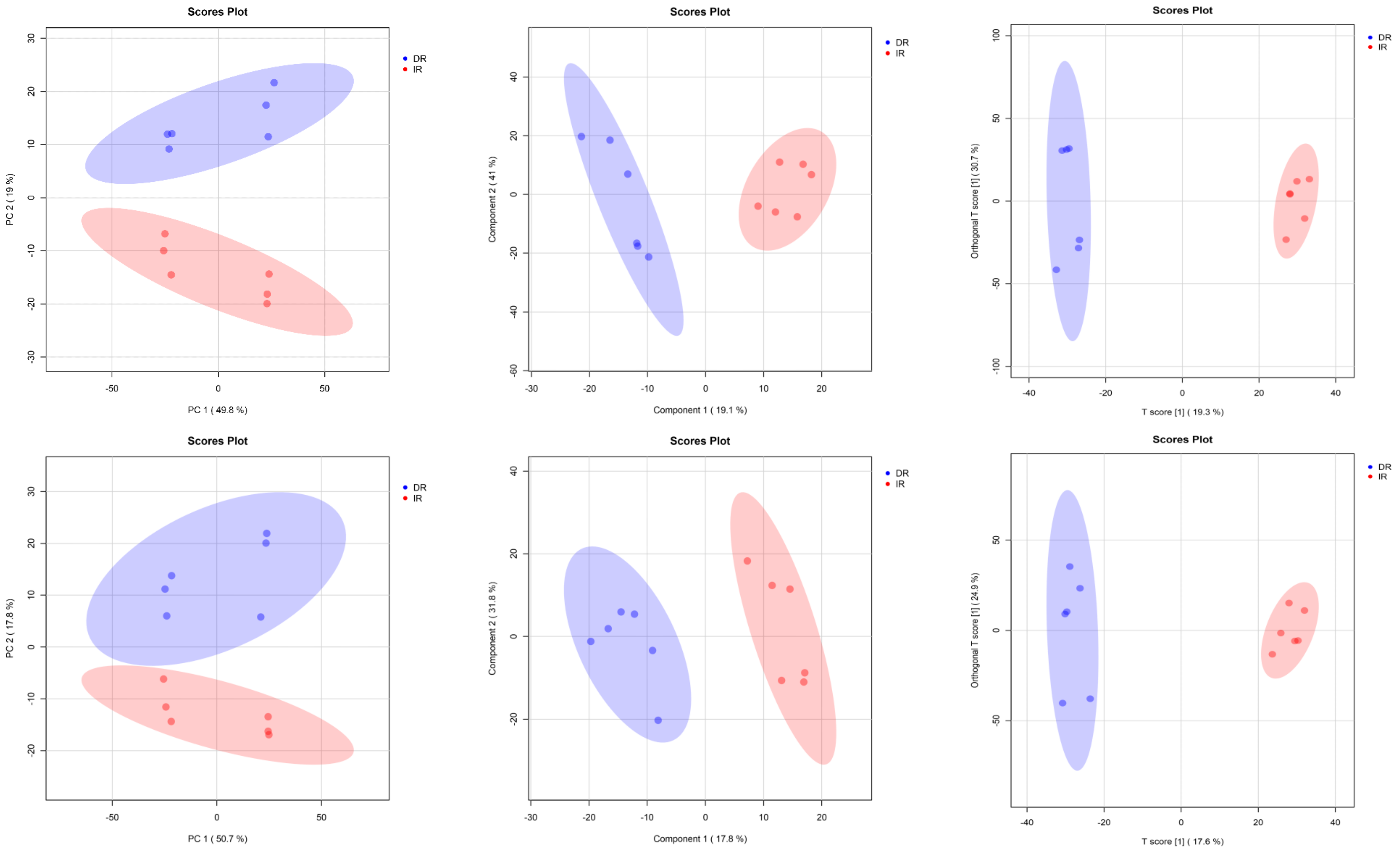

Supplementary Figure 2-1. PCA, PLS-DA, and OPLS-DA (left to right) for Drought versus Irrigated samples for 3 replicates via two ion modes, negative (top) and positive (bottom).

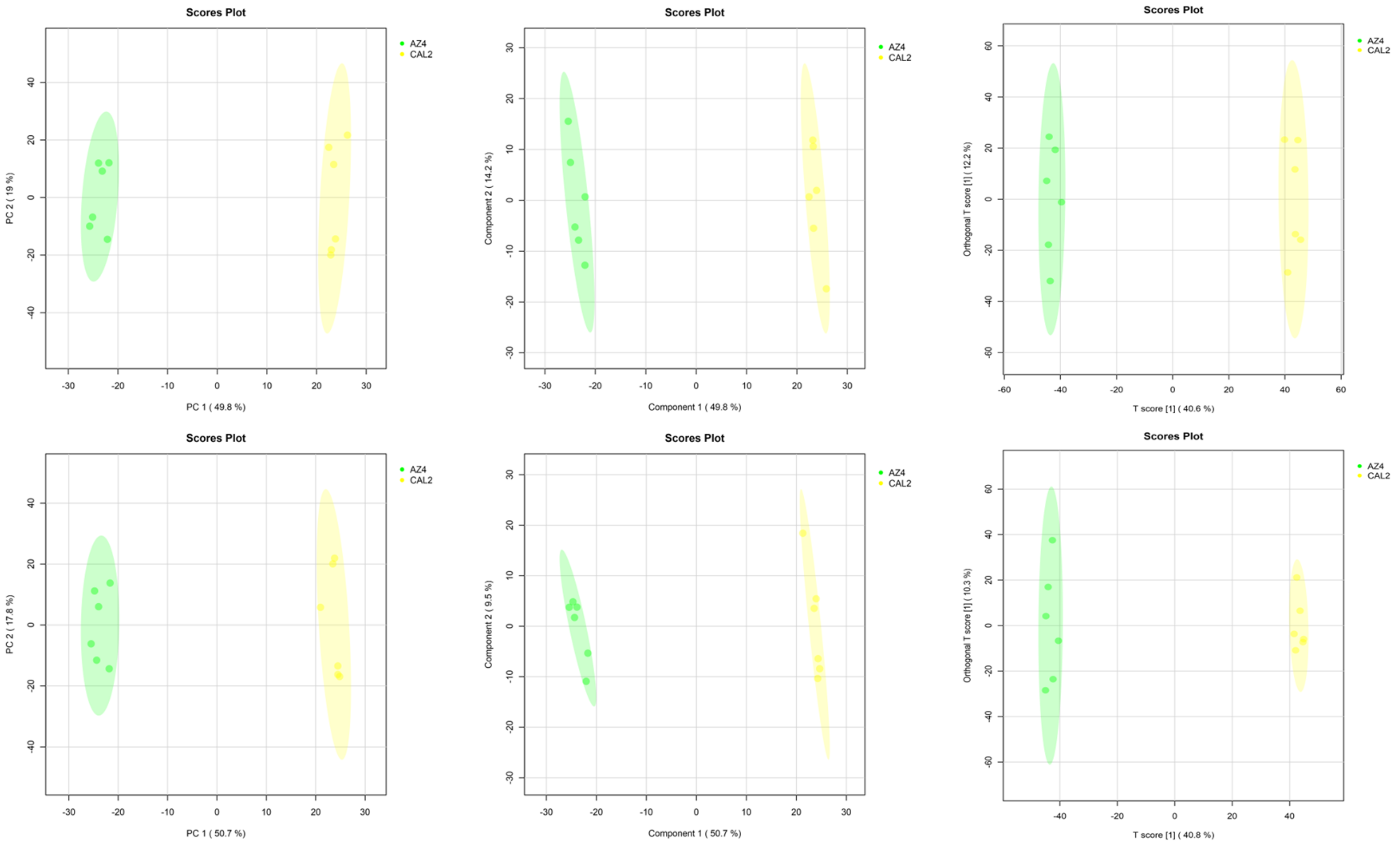

Supplementary Figure 2-2. PCA, PLS-DA, and OPLS-DA (left to right) for AZ-4 versus CAL-2 samples for 3 replicates via two ion modes, negative (top) and positive (bottom).

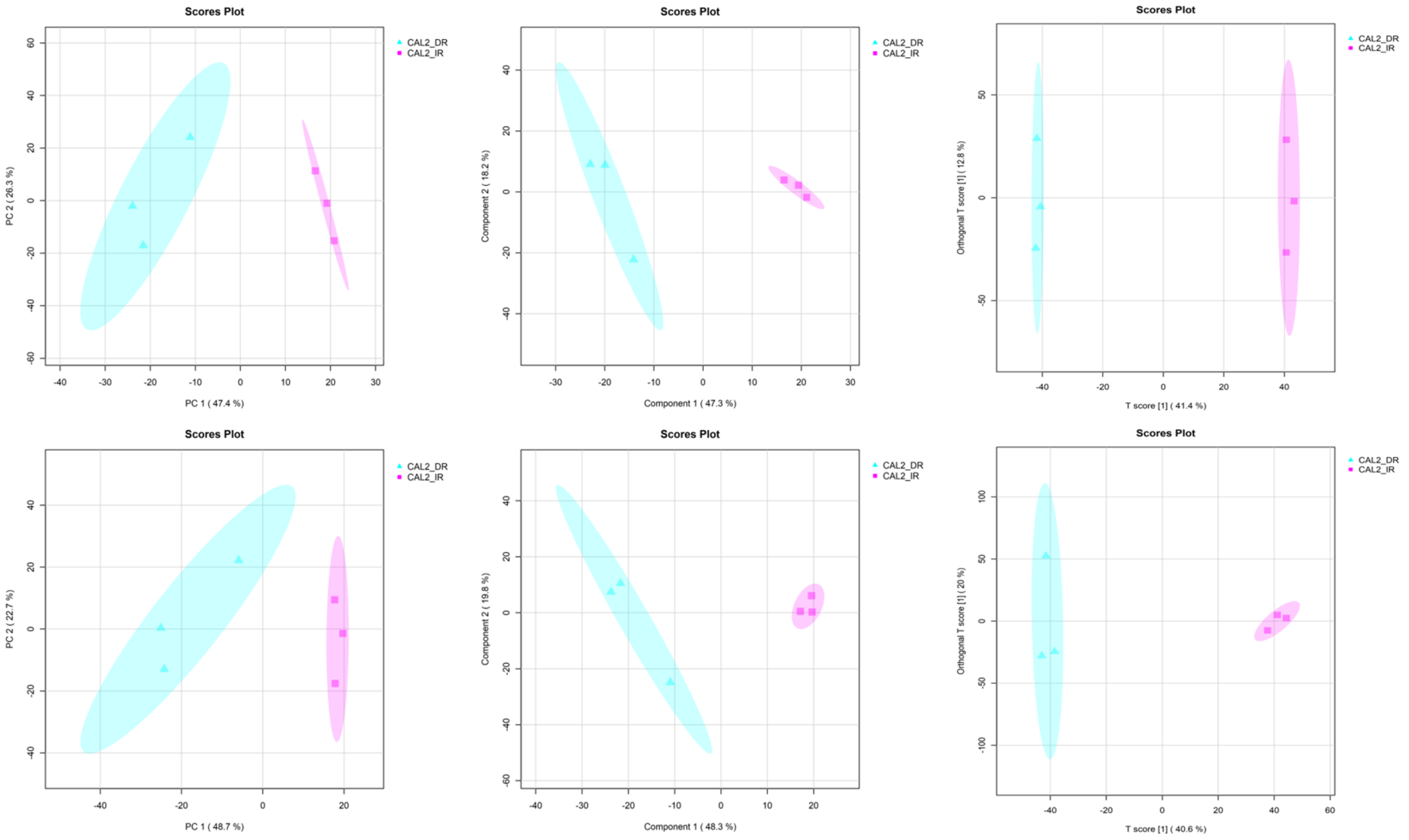

Supplementary Figure 2-3. PCA, PLS-DA, and OPLS-DA (left to right) for CAL-2 samples under Drought versus Irrigated for 3 replicates via two ion modes, negative (top) and positive (bottom).

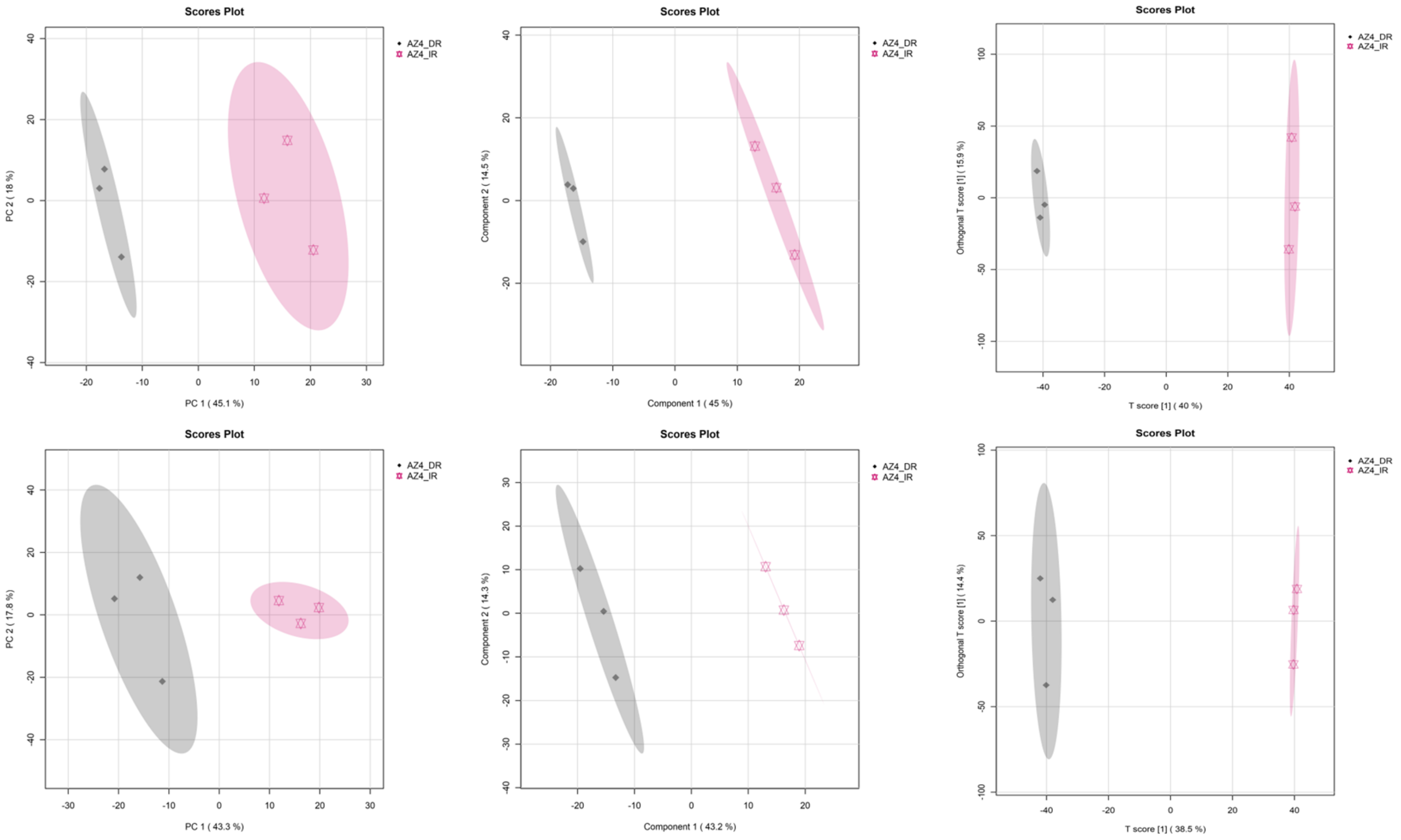

Supplementary Figure 2-4. PCA, PLS-DA, and OPLS-DA (left to right) for AZ-4 samples under Drought versus Irrigated for 3 replicates via two ion modes, negative (top) and positive (bottom).

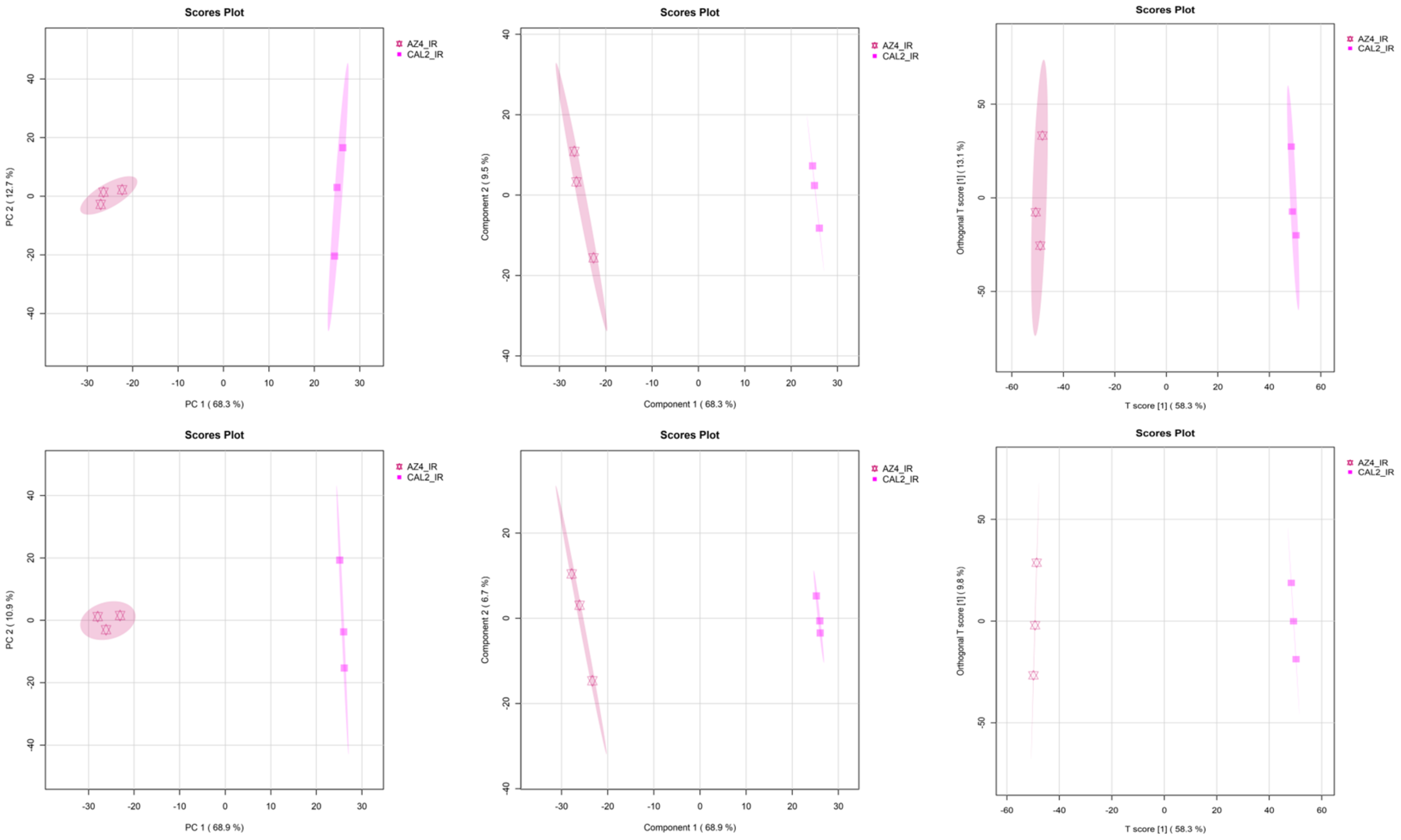

Supplementary Figure 2-5. PCA, PLS-DA, and OPLS-DA (left to right) for AZ-4 Irrigated samples versus CAL-2 Irrigated for 3 replicates via two ion modes, negative (top) and positive (bottom).

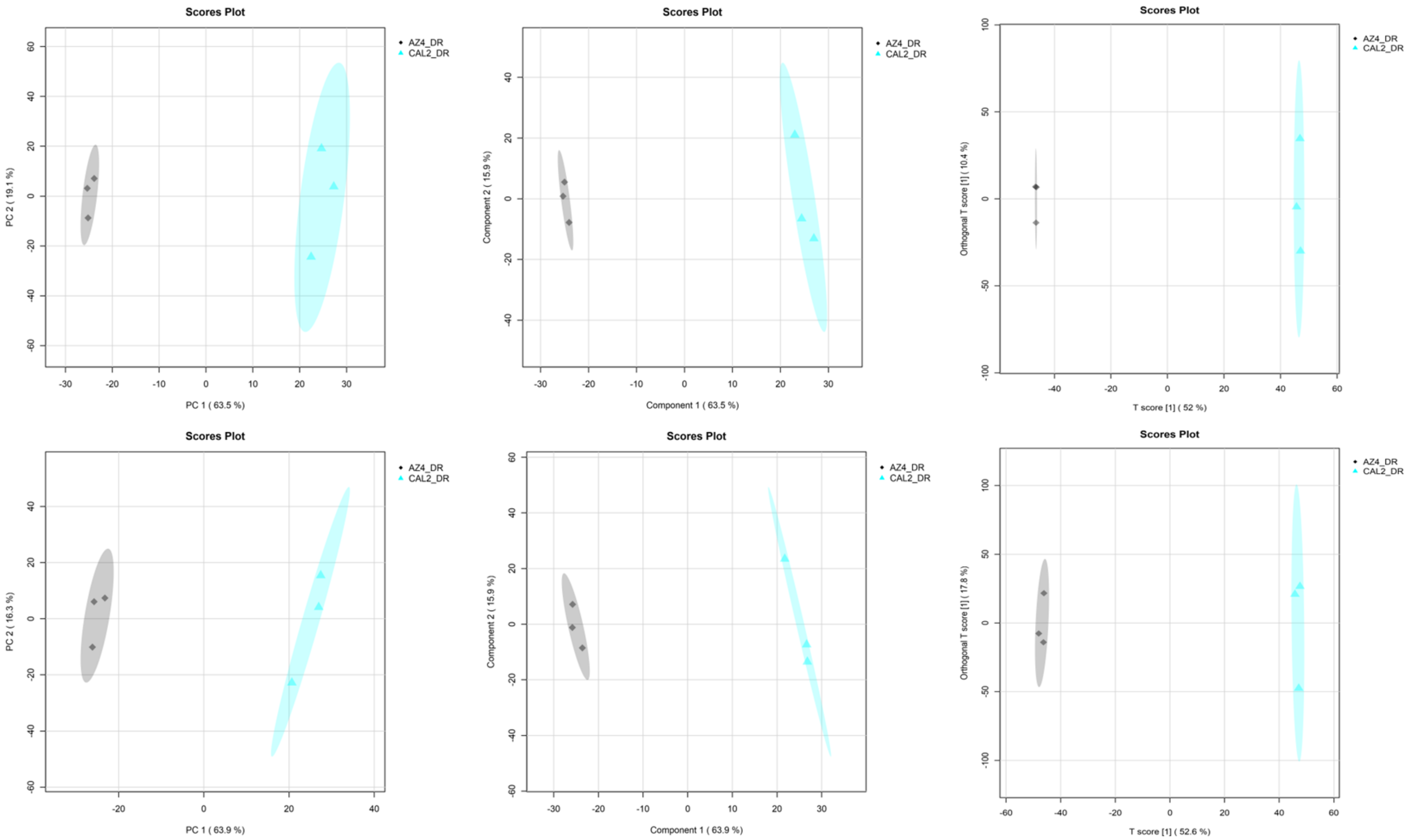

Supplementary Figure 2-6. PCA, PLS-DA, and OPLS-DA (left to right) for AZ-4 Drought samples versus CAL-2 Drought for 3 replicates via two ion modes, negative (top) and positive (bottom).
