# Supplementary material for: Metabolomics reveal drought-stress responses in guayule, a semi-arid rubber crop
Source: Metabolomics. 2026 Jun 16;22(4):97. doi: 10.1007/s11306-026-02487-5 (PMC13272222; doi:10.1007/s11306-026-02487-5)
Supplement: Supplementary file 2 — Supplementary file2 (DOCX 37 KB) [file 11306_2026_2487_MOESM2_ESM.docx]

**Supplementary Table 1. Top five metabolites between drought and irrigated AZ-4 and CAL-2 side-by-side via two analyses.** (−) sign notes for depletion and (+) sign notes for accumulation; yellow: metabolites overlapped between two analyses. Full list of metabolites can be found in Supplementary Table 10 for AZ-4, and Supplementary Table 11 for CAL-2.

| Cultivar | Top Metabolites | Analysis | Log2(Fold Change) | −Log10(p-value) |
| --- | --- | --- | --- | --- |
| AZ-4 | 2-dodecylbenzenesulfonic acid | Raw | −3.24 | 0.74 |
|  | Spinacoside D |  | −3.02 | 0.54 |
|  | Prostaglandin lactone-diol |  | −2.86 | 0.44 |
|  | Catechin 3'-glucuronide |  | −2.58 | 1.14 |
|  | 3-Oxo-4,6-choladienoic acid |  | −2.55 | 0.47 |
|  | Cyclomulberrin |  | +3.90 | 1.19 |
|  | Ornithine |  | +3.84 | 0.76 |
|  | Arginine |  | +3.65 | 0.81 |
|  | Monoacylglycerol(MG)(20:3(6,8,11)-OH(5)/0:0/0:0) |  | +3.39 | 1.16 |
|  | Uric acid |  | +3.14 | 0.55 |
|  | 4'-Phosphopantothenoylcysteine | Hierarchical Cluster Analysis | −2.10 | 1.34 |
|  | N-Carbamoylcytidine |  | −1.91 | 1.48 |
|  | Caffeoyl C1-glucuronide |  | −1.61 | 1.40 |
|  | Pelargonidin 3-rhamnoside |  | −1.33 | 1.35 |
|  | 6-Desmethylmonacolin J |  | −1.27 | 1.35 |
|  | Cornoside |  | +1.70 | 1.43 |
|  | Methyl salicylate O-[rhamnosyl-(1->6)-glucoside] |  | +1.64 | 1.53 |
|  | 5-p-Coumaroylquinic acid |  | +1.45 | 1.53 |
|  | Methyl 7-epi-12-hydroxyjasmonate glucoside |  | +1.45 | 1.39 |
|  | N,N'-diacetylchitobiose |  | +1.28 | 1.32 |
| CAL-2 | 9-Methyluric acid | Raw | −5.73 | 0.47 |
|  | 7a,12a-Dihydroxy-3-oxo-4-cholenoic acid |  | −4.15 | 0.43 |
|  | 4'-Phosphopantothenoylcysteine |  | −3.78 | 1.46 |
|  | Lenalidomide |  | −3.63 | 1.37 |
|  | 4-Amino-3-hydroxybutyrate |  | −3.30 | 0.73 |
|  | Oleragenoside |  | +5.34 | 0.78 |
|  | Licoricesaponin B2 |  | +5.11 | 0.77 |
|  | Licoricesaponin J2 |  | +4.98 | 0.78 |
|  | Momordin I |  | +4.87 | 0.56 |
|  | Azukisaponin III |  | +4.81 | 0.67 |
|  | 4'-Phosphopantothenoylcysteine | Hierarchical Cluster Analysis | −3.78 | 1.46 |
|  | Lenalidomide |  | −3.63 | 1.37 |
|  | 2-Dodecylbenzenesulfonic acid |  | −3.29 | 1.65 |
|  | PE(6 keto-PGF1alpha/18:0) |  | −2.94 | 1.39 |
|  | Ribavirin monophosphate |  | −2.82 | 1.77 |
|  | Dihydrolipoamide |  | +2.77 | 1.62 |
|  | Oleoside dimethyl ester |  | +2.45 | 1.37 |
|  | 13-HDoHE |  | +2.40 | 1.69 |
|  | 4'-Methyl-epigallocatechin 7-glucuronide |  | +2.09 | 1.42 |
|  | Camellianin A |  | +1.95 | 1.49 |

**Supplementary Table 2. Top enriched pathways** **between drought and irrigated AZ-4 and CAL-2 side-by-side via Correlation Network Analysis.** Full list of pathways can be found in Supplementary Table 10 for AZ-4, and Supplementary Table 11 for CAL-2.

| Cultivar | Top Pathways | Analysis | −Log10(p-value) |
| --- | --- | --- | --- |
| AZ-4 | Amino Sugar Metabolism | Correlation Network Analysis | 2.87 |
|  | Pantothenate and CoA Biosynthesis |  | 3.35 |
| CAL-2 | Ubiquinone Biosynthesis |  | 4.21 |
|  | Beta-Alanine Metabolism |  | 4.00 |
|  | Phenylalanine and Tyrosine Metabolism |  | 4.00 |
|  | Ammonia Recycling |  | 4.00 |
|  | Cysteine Metabolism |  | 4.00 |

**Supplementary Table 3. Top five metabolites between drought AZ-4 and drought CAL-2 side-by-side via two analyses.** Yellow: metabolites overlapped between two analyses. Full list of metabolites can be found in Supplementary Table 12.

| Top Metabolites | Analysis | Dominant in Cultivar | Log2(Fold Change) | −Log10(p-value) |
| --- | --- | --- | --- | --- |
| 2-Aminophenol N-formate sulfate | Raw | AZ-4 | 9.82 | 1.80 |
| PIP(PGF1alpha/16:2) |  |  | 7.35 | 2.40 |
| Apitolisib |  |  | 7.15 | 2.36 |
| Lysyl-Phenylalanine |  |  | 6.89 | 1.48 |
| 5-Imino-2-methyl-1-cyclopenten-1-ol |  |  | 6.84 | 2.68 |
| 4'-Methyl-epigallocatechin 7-glucuronide |  | CAL-2 | 7.20 | 1.93 |
| Kiwiionoside |  |  | 7.17 | 1.56 |
| Mirificin |  |  | 7.11 | 1.40 |
| Prostaglandin lactone-diol |  |  | 6.81 | 0.92 |
| Paucin |  |  | 6.57 | 1.52 |
| 2-Aminophenol N-formate sulfate | Hierarchical Cluster Analysis | AZ-4 | 9.82 | 1.80 |
| PIP(PGF1alpha/16:2) |  |  | 7.35 | 2.40 |
| Apitolisib |  |  | 7.15 | 2.36 |
| Lysyl-Phenylalanine |  |  | 6.89 | 1.48 |
| 5-Imino-2-methyl-1-cyclopenten-1-ol |  |  | 6.84 | 2.68 |
| 4'-Methyl-epigallocatechin 7-glucuronide |  | CAL-2 | 7.20 | 1.93 |
| Kiwiionoside |  |  | 7.17 | 1.56 |
| Mirificin |  |  | 7.11 | 1.40 |
| Paucin |  |  | 6.81 | 0.92 |
| Rheinoside C |  |  | 6.57 | 1.52 |

**Supplementary Table 4. All enriched pathways between drought AZ-4 and drought CAL-2 side-by-side via Correlation Network Analysis.** Full list of pathways can be found in Supplementary Table 12.

| Top Pathways | Analysis | Dominant in Cultivar | −Log10(p-value) |
| --- | --- | --- | --- |
| Tyrosine Metabolism | Correlation Network Analysis | AZ-4 | 3.06 |
| Tryptophan Metabolism |  |  | 2.81 |
| Arginine and Proline Metabolism |  |  | 2.68 |
| Pantothenate and CoA Biosynthesis |  |  | 1.99 |
| Estrone Metabolism |  | CAL-2 | 3.48 |
| Ubiquinone Biosynthesis |  |  | 2.92 |
| Pyrimidine Metabolism |  |  | 2.68 |
| Amino Sugar Metabolism |  |  | 2.43 |
| Steroidogenesis |  |  | 1.72 |

**Supplementary Table 5. Top five metabolites between AZ-4 CAL-2 regardless of treatments side-by-side via two analyses.** Yellow: metabolites overlapped between two analyses. Full list of metabolites can be found in Supplementary Table 13.

| Top Metabolites | Analysis | Dominant in Cultivar | Log2(Fold Change) | −Log10(p-value) |
| --- | --- | --- | --- | --- |
| 2-Aminophenol N-formate sulfate | Raw | AZ-4 | 9.86 | 3.66 |
| 7'-O-Methylmarmin |  |  | 7.21 | 2.64 |
| Apitolisib |  |  | 7.17 | 3.92 |
| PIP(PGF1alpha/16:2) |  |  | 7.12 | 2.20 |
| Lysyl-Phenylalanine |  |  | 7.00 | 2.10 |
| Kiwiionoside |  | CAL-2 | 7.33 | 3.54 |
| Mirificin |  |  | 7.06 | 2.69 |
| 4'-Methyl-epigallocatechin 7-glucuronide |  |  | 6.82 | 1.71 |
| Rheinoside C |  |  | 6.41 | 2.55 |
| Histidylaspartic acid |  |  | 6.26 | 2.21 |
| 2-Aminophenol N-formate sulfate | Hierarchical Cluster Analysis | AZ-4 | 9.86 | 3.66 |
| 7'-O-Methylmarmin |  |  | 7.21 | 2.64 |
| Apitolisib |  |  | 7.17 | 3.92 |
| PIP(PGF1alpha/16:2) |  |  | 7.12 | 2.20 |
| Lysyl-Phenylalanine |  |  | 7.00 | 2.10 |
| Kiwiionoside |  | CAL-2 | 7.33 | 3.54 |
| Mirificin |  |  | 7.06 | 2.69 |
| 4'-Methyl-epigallocatechin 7-glucuronide |  |  | 6.82 | 1.71 |
| Rheinoside C |  |  | 6.41 | 2.55 |
| Histidylaspartic acid |  |  | 6.26 | 2.21 |

**Supplementary Table 6. Top five enriched pathways between AZ-4 CAL-2 regardless of treatments side-by-side via Correlation Network Analysis.** Full list of pathways can be found in Supplementary Table 13.

| Top Pathways | Analysis | Dominant in Cultivar | −Log10(p-value) |
| --- | --- | --- | --- |
| Tryptophan Metabolism | Correlation Network Analysis | AZ-4 | 6.57 |
| Tyrosine Metabolism |  |  | 5.86 |
| Ubiquinone Biosynthesis |  | CAL-2 | 7.86 |
| Estrone Metabolism |  |  | 7.25 |
| Amino Sugar Metabolism |  |  | 6.19 |

**Supplementary Table 7. 53 filtered metabolites from drought versus irrigated conditions based on significance and fold changes from 1927 significant metabolites found via positive and negative ion mode into 6 pathway categories.** (−) sign notes for depletion and (+) sign notes for accumulation. Descriptions obtained from Plant Metabolic Network (Hawkins et al., 2025) and RefMetaPlant (Shi et al., 2024).

| **Metabolite** | **Category** | **Log2(Fold Change)** | **Description** |
| --- | --- | --- | --- |
| 4-amino-3-hydroxybutyrate | Precursors of Resin and Rubber Biosynthesis | −3.30 | Amino acid derivative from amino acid metabolism; may support biosynthesis |
| 4'-phosphopantothenoylcysteine |  | −3.78 | Precursor to Coenzyme A, essential for fatty acid and isoprenoid biosynthesis. |
| 4-trimethylammoniobutanoic acid |  | −1.84 | Betaine-like compound, acts as an osmoprotectant and supports stress-linked biosynthesis. |
| Arginine |  | +3.65 | Amino acid involved in nitrogen metabolism; may support precursor synthesis. |
| Dihydrolipoamide |  | +2.77 | Cofactor in acetyl-CoA production, which feeds into isoprenoid synthesis. |
| Glutamic acid |  | −1.37 | Central amino acid in nitrogen metabolism; supports biosynthetic pathways. |
| Monoacylglycerol(MG)(20:3-OH/0:0/0:0) |  | +3.80 | Lipid precursor that may contribute to membrane or rubber-related lipid synthesis. |
| N,N'-diacetylchitobiose |  | +1.28 | Disaccharide possibly involved in structural polysaccharide metabolism. |
| Ornithine |  | +3.84 | Urea cycle intermediate, precursor to polyamines and nitrogen donors. |
| Proline |  | +2.1 | Amino acid with osmoprotective properties; supports biosynthesis under stress. |
| Ubiquinone-1 |  | −2.07 | Isoprenoid derivative structurally related to polyisoprenes; may share biosynthetic origin. |
| Azukisaponin III | Product, Byproduct, or Parallel Product of Resin and Rubber Biosynthesis | +4.81 | Saponin found in latex-producing plants; may be co-extracted with rubber. |
| Cyclomulberrin |  | +3.9 | Flavonoid from mulberry, a latex-producing genus; potential byproduct. |
| Dihydro-alpha-santalic acid |  | +0.43 | Sesquiterpene possibly derived from terpenoid biosynthesis in rubber-producing tissues. |
| Licoricesaponin-B2 |  | +5.11 | Saponin from licorice, often found in latex-rich plants. |
| Licoricesaponin-J2 |  | +4.5 | Similar to Licoricesaponin-B2; triterpenoid saponin. |
| Masticadienonic acid |  | −1.19 | Triterpenoid resin acid found in latex-producing plants. |
| Momordin I |  | +4.87 | Triterpenoid saponin associated with latex-rich plant species. |
| Myzodendrone |  | +1.33 | Phenolic compound found in parasitic plants; may be latex-associated. |
| Oleoside dimethyl ester |  | +2.45 | Iridoid glycoside found in latex-producing plants. |
| Oleragenoside |  | +5.34 | Iridoid glycoside similar to oleoside; latex-related. |
| 13-HDoHE | Stress Signaling Molecules | +2.4 | Hydroxylated derivative of docosahexaenoic acid (DHA); lipid mediator involved in oxidative stress and inflammation |
| 3-oxo-4,6-choladienoic acid |  | −2.55 | Bile acid intermediate; may act in signaling or detoxification. |
| 5-acetylamino-6-formylamino-3-methyluracil |  | −1.95 | Modified nucleotide; may be involved in stress-related RNA metabolism. |
| 6-desmethylmonacolin J |  | −1.85 | Statin precursor; modulates lipid metabolism and stress responses. |
| 7a,12a-dihydroxy-3-oxo-4-cholenoic acid |  | −4.15 | Bile acid derivative; involved in metabolic signaling. |
| Cornoside |  | +1.96 | Iridoid glycoside with anti-inflammatory and stress-related activity. |
| LysoPC(18:3/0:0) |  | −1.05 | Lysophospholipid; involved in membrane remodeling and stress signaling. |
| LysoPE(18:3/0:0) |  | −1.18 | Similar to LysoPC; lipid signaling molecule. |
| Methyl 7-epi-12-hydroxyjasmonate glucoside |  | +1.45 | Jasmonate derivative; key plant stress hormone. |
| Methyl salicylate O-[rhamnosyl-(1->6)-glucoside] |  | +1.64 | Salicylate conjugate; plant defense signal. |
| N-carbamoylcytidine |  | −2.30 | Modified nucleotide; may play a role in stress adaptation. |
| PE(6 keto-PGF1alpha/18:0) |  | −2.94 | Prostaglandin-containing phospholipid; inflammatory signal. |
| PIP(PGD2/18:0) |  | +2.36 | Prostaglandin D2-containing lipid; immune and stress mediator. |
| Prostaglandin lactone-diol |  | −2.86 | Prostaglandin derivative; involved in inflammation and stress. |
| 4'-methyl-epigallocatechin 7-glucuronide | Antioxidant and Defense | +2.09 | Flavonoid conjugate with antioxidant properties. |
| 5-p-coumaroylquinic acid |  | +1.45 | Phenolic ester; antioxidant and structural component in lignin biosynthesis |
| 9-hydroxy-4-methoxypsoralen 9-glucoside |  | −1.11 | Coumarin derivative; photoreactive compound with antioxidant and stress-related roles |
| 9-methyluric acid |  | −5.73 | Derivative of uric acid; retains antioxidant activity. |
| Caffeoyl C1-glucuronide |  | −2.09 | Phenolic acid conjugate; antioxidant and anti-inflammatory. |
| Camellianin A |  | +1.95 | Flavonoid glycoside with strong antioxidant activity. |
| Catechin 3'-glucuronide |  | −2.58 | Flavonoid metabolite; potent antioxidant. |
| Coniferin |  | +1.14 | Lignan precursor with antioxidant and structural roles. |
| Feruloylquinic acid |  | +1.44 | Ester of ferulic acid; antioxidant and UV-protective. |
| Pelargonidin 3-rhamnoside |  | −1.9 | Anthocyanin pigment with antioxidant properties. |
| Salviaflaside |  | −2.16 | Flavonoid glycoside with antioxidant and anti-inflammatory effects. |
| Spinacoside-D |  | −3.02 | Glycoside with antioxidant potential, found in spinach. |
| Stizolobic acid |  | −1.83 | Amino acid derivative with antioxidant and stress-protective roles. |
| Uric acid |  | +3.14 | Antioxidant that scavenges reactive oxygen species. |
| 2-dodecylbenzenesulfonic acid | True Synthetic and Contaminants | −3.29 | Synthetic surfactant; exogenous compound used in latex processing |
| Lenalidomide |  | −2.68 | Immunomodulatory drug; influences cellular stress and immune signaling. |
| Ribavirin monophosphate |  | −2.82 | Antiviral nucleotide; modulates immune stress responses. |
| Tetrahydrocortisone |  | −0.51 | Corticosteroid; regulates stress and immune responses. |

**Supplementary Table 8. 27 filtered metabolites from AZ-4 versus CAL-2 based on significance and fold changes into 6 pathway categories. (−) sign notes for higher concentration in CAL-2 and (+) sign notes for higher concentration in AZ-4.** Descriptions obtained from Plant Metabolic Network (Hawkins et al., 2025) and RefMetaPlant (Shi et al., 2024).

| **Metabolite** | **Category** | **Log2(Fold Change)** | **Description** |
| --- | --- | --- | --- |
| 3-pyridylacetic acid | Precursors of Rubber Biosynthesis | +4.76 | A small organic acid that may participate in nitrogen metabolism linked to secondary biosynthesis. |
| 5-imino-2-methyl-1-cyclopenten-1-ol |  | +6.84 | A cyclopentene derivative potentially involved in early terpenoid or isoprene unit formation. |
| Histidylaspartic acid |  | −6.56 | A peptide possibly involved in enzymatic regulation or precursor signaling. |
| Kiwiionoside |  | −7.47 | A glycoside that may be part of upstream metabolic flux toward rubber biosynthesis. |
| Lysyl-phenylalanine |  | +7.30 | A dipeptide that could contribute to amino acid-derived biosynthetic intermediates. |
| Mono(glucosyluronic acid)bilirubin | Product, Byproduct, or Parallel Product of Rubber Biosynthesis | +2.35 | A bilirubin conjugate that could be excreted alongside rubber-related metabolic waste. |
| Monoacylglycerol(MG)(0:0/5-iso PGF2VI/0:0) |  | +3.10 | A lipid signaling molecule that may arise as a byproduct of polyisoprene metabolism. |
| PIP(PGF1alpha/16:2) |  | +7.35 | A prostaglandin-like compound possibly co-produced during lipid-based biosynthesis. |
| Prostaglandin lactone-diol |  | −6.81 | A cyclic prostaglandin derivative that may reflect parallel lipid processing in rubber-producing tissues. |
| Rheinoside C |  | −6.5 | A plant glycoside that may accumulate in latex-rich tissues as a secondary metabolite. |
| 2-aminophenol N-formate sulfate | Stress Signaling Molecules | +9.92 | A nitrogen-containing compound possibly involved in oxidative or pathogen-triggered signaling. |
| 7'-O-methylmarmin |  | +7.60 | A coumarin derivative that may act as a signal in response to biotic or abiotic stress. |
| Citrusin B |  | +2.20 | A limonoid involved in plant defense and stress-induced secondary metabolism. |
| Mirificin |  | −7.11 | A phytoestrogen-like compound that could modulate hormonal or environmental stress responses. |
| Sampatrilat |  | +3.42 | A vasopeptidase inhibitor that may mimic or interfere with peptide-based stress signaling. |
| 4'-methyl-epigallocatechin 7-glucuronide | Antioxidant and Defense | −7.2 | A flavonoid glucuronide with strong antioxidant and anti-inflammatory activity. |
| 6-dehydrotestosterone glucuronide |  | +6.17 | (Not previously listed—please confirm if this belongs in your dataset.) |
| 6-methoxyluteolin 7-glucuronide |  | +4.79 | A methoxylated flavone that helps mitigate oxidative stress in plant tissues. |
| Calendoflaside |  | −2.26 | A saponin-like compound that enhances plant immunity. |
| Chalcomoracin |  | +3.10 | A prenylated flavonoid with antimicrobial and antioxidant effects. |
| Isoeriocitrin |  | −1.85 | A citrus-derived flavonoid with potent radical-scavenging properties. |
| Kaempferol 3-rhamnosyl-(1->3)-(4'''-acetylrhamnosyl)(1->6)-glucoside |  | +5.76 | A complex flavonoid glycoside contributing to pathogen resistance. |
| Myricanol 5-[arabinosyl-(1->6)-glucoside] |  | +2.28 | A glycosylated diarylheptanoid with anti-inflammatory and defense roles. |
| Ononin |  | −3.66 | An isoflavone glycoside with antioxidant and protective functions. |
| Apitolisib | True Synthetic and Contaminants | +7.19 | A synthetic PI3K/mTOR inhibitor used in cancer research, not naturally found in plants. |
| Paucin |  | −6.57 | A rare or poorly characterized compound likely synthetic or exogenous to plant metabolism. |
| Tetramethylchromanol glucoside |  | +3.05 | A synthetic antioxidant derivative possibly introduced through contamination. |
